# Novel principles of gamma-retroviral insertional transcription activation in murine leukemia virus-induced end-stage tumors

**DOI:** 10.1186/1742-4690-11-36

**Published:** 2014-05-19

**Authors:** Martin Sokol, Matthias Wabl, Irene Rius Ruiz, Finn Skou Pedersen

**Affiliations:** 1 Department of Molecular Biology and Genetics, Aarhus University, DK-8000 Aarhus, Denmark; 2 Department of Microbiology and Immunology, University of California, San Francisco, CA 94143, USA

**Keywords:** Gamma-retrovirus, Insertional mutagenesis, Oncogenesis, Deep sequencing, Chromatin immunoprecipication with sequencing (ChIP-seq), Retroviral integration sites, RNA sequencing (RNA-seq)

## Abstract

**Background:**

Insertional mutagenesis screens of retrovirus-induced mouse tumors have proven valuable in human cancer research and for understanding adverse effects of retroviral-based gene therapies. In previous studies, the assignment of mouse genes to individual retroviral integration sites has been based on close proximity and expression patterns of annotated genes at target positions in the genome. We here employed next-generation RNA sequencing to map retroviral-mouse chimeric junctions genome-wide, and to identify local patterns of transcription activation in T-lymphomas induced by the murine leukemia gamma-retrovirus SL3-3. Moreover, to determine epigenetic integration preferences underlying long-range gene activation by retroviruses, the colocalization propensity with common epigenetic enhancer markers (H3K4Me1 and H3K27Ac) of 6,117 integrations derived from end-stage tumors of more than 2,000 mice was examined.

**Results:**

We detected several novel mechanisms of retroviral insertional mutagenesis: bidirectional activation of mouse transcripts on opposite sides of a provirus including transcription of unannotated mouse sequence; sense/antisense-type activation of genes located on opposite DNA strands; tandem-type activation of distal genes that are positioned adjacently on the same DNA strand; activation of genes that are not the direct integration targets; combination-type insertional mutagenesis, in which enhancer activation, alternative chimeric splicing and retroviral promoter insertion are induced by a single retrovirus. We also show that irrespective of the distance to transcription start sites, the far majority of retroviruses in end-stage tumors colocalize with H3K4Me1 and H3K27Ac-enriched regions in murine lymphoid tissues.

**Conclusions:**

We expose novel retrovirus-induced host transcription activation patterns that reach beyond a single and nearest annotated gene target. Awareness of this previously undescribed layer of complexity may prove important for elucidation of adverse effects in retroviral-based gene therapies. We also show that wild-type gamma-retroviruses are frequently positioned at enhancers, suggesting that integration into regulatory regions is specific and also subject to positive selection for sustaining long-range gene activation in end-stage tumors. Altogether, this study should prove useful for extrapolating adverse outcomes of retroviral vector therapies, and for understanding fundamental cellular regulatory principles and retroviral biology.

## Background

The murine leukemia viruses (MLVs) are slowly-transforming gamma-retroviruses that induce tumors of hematopoietic origin upon injection into susceptible newborn mice [1,2]. MLVs are simple retroviruses that contain only the *gag*, *pol* and *env* genes flanked at both ends by major regulatory regions, the long terminal repeats (LTRs) composed of U3, R and U5. A hallmark of retroviral replication is the stable integration of the ~9 kb genome into host chromosomes as a provirus which makes gamma-retroviral and lentiviral-based vectors attractive gene delivery vehicles for some therapeutic applications in human gene therapy ([3-6] for recent reviews). MLVs alter mouse gene expression via insertional mutagenesis (IM), which induces transcriptional or post-transcriptional deregulation of affected genes. The major determinants of retroviral IM are the LTRs, which contain viral promoter and enhancer elements in U3. Insertion of the provirus upstream from the first exon or in early introns may induce transcription of the target mouse gene driven by the retroviral promoter and this is known as promoter insertion. Transcripts may also undergo alternative splicing resulting from the use of either proviral or cryptic intronic splice sites, while integration in the 3′-end of a gene may result in truncation of the native transcript due to usage of a proviral poly (A) signal present in the LTR [3-7]. MLV enhancer activation mutagenesis is induced by enhancer elements present in U3, which augment transcription from cellular promoters over long distances by recruitment of transcription factors. IM screens based on retroviral and transposon mouse models have proven an effective approach in identifying human cancer genes [3-6,8,9].

IM has received increased attention due to the occurrence of adverse events following gamma-retroviral vector-based gene therapy to correct the X-linked severe combined immunodeficiency disease (X-SCID) in which a lymphoproliferative disorder was induced in patients by activation of the *LMO2* oncogene [10-13]. In another clinical trial to correct the X-linked chronic granulomatous disease (X-CGD) immunodeficiency, gamma-retroviral vector integration caused activation of *MDS1-EVI1*, resulting in myelodysplastic syndrome (MDS) with transition to acute myeloid leukemia (AML) and development of monosomy 7 [14,15]. Therefore, understanding fundamental principles of retrovirus-induced tumorigenesis including integration site preferences and mechanisms of host sequence deregulation is vital for the improvement of therapy safety and assessment of possible genetic disruptions in the course of treatment.

While earlier studies suggested that MLVs preferentially target DNase I hypersensitive regions, transcription start sites (TSSs) and CpG islands [16,17], several *in vitro* studies and studies of cultured primary cells have shown that gamma-retroviral vectors favor integration into nucleosomal DNA [18-24]. Chromatin associated with histone methylations and acetylations including H3K4Me3 and H3K27Ac/H3K4Me1 which are common promoter and enhancer markers, respectively [25-28], are major targets of gamma-retroviral vector integration [22-24].

The bromodomain and extraterminal (BET) proteins mediate MLV integration at TSSs by tethering the integrase to acetylated H3 and H4 tails [29], and expression of a BET fusion protein containing the chromatin binding domain of the lentiviral integrase cofactor LEDGF/p75 results in retargeting of MLV to match the integration profile of HIV [30]. While promoters and promoter-proximal enhancers are functionally similar [31,32], promoter-distal enhancers may enhance transcription from thousands of bp away. One example is the distal limb bud enhancer of mouse *Shh* which is positioned 1 Mb from the *Shh* promoter [33,34]. The mammalian genome is demarcated into coregulated enhancer and promoter units known as EPUs [25], and it has been shown that proviruses positioned in *c-Myb* upstream elements that coincide with enhancers in the EPU of this gene, establish physical contact with the *c-Myb* promoter through DNA looping [35]. Taken together, this suggests that the three-dimensional structure of the genome influences promoter-distal integration-mediated long-range gene activation. It is currently not known if integration outside TSSs is mediated by BET proteins or other factors.

In the past, MLV integrations were identified using anchored-type PCR methods and Sanger sequencing, including e.g. a special 5′-end primer called a splinkerette, or inverse PCR [36-38]. Next-generation sequencing (NGS) has proven effective in the analysis of retroviral biology in diverse contexts, including HIV-1 infection and gamma-retroviral and lentiviral vector systems, and is commonly coupled with anchored-type PCR to determine positions of retroviral integration in DNA [39-46].

In this study we subjected four NMRI mouse tumors induced upon infection with the rapid T-lymphomagenic MLV SL3-3 wild-type strain to strand-specific and paired-end RNA sequencing (RNA-seq) to determine retroviral integrations genome-wide, and to identify local patterns of retroviral IM. We then used a dataset of 6,117 SL3-3 integrations derived from lymphoid tumors of more than 2,000 mice to determine the colocalization propensity of proviruses with ENCODE immunoprecipitation with sequencing (ChIP-seq) enhancer and promoter markers *in vivo*[25]. Our study unravels novel mechanisms of retroviral IM involving bidirectional and tandem-type activation patterns, as well as more complex patterns including activation of major unannotated transcripts and combination-type activation where transcription is induced by promoter insertion, chimeric alternative splicing and enhancer activation by a single provirus. We also show that the majority of proviruses in tumors are located at ChIP-seq H3K4Me1/H3K27Ac-enriched positions irrespective of their distance to TSSs, suggesting that insertion into cellular regulatory regions is highly specific and subject to positive selection during tumorigenesis for sustaining long-range gene activation.

## Results

### Chimeric sequencing reads expose retroviral integration sites

**Figure 1:**
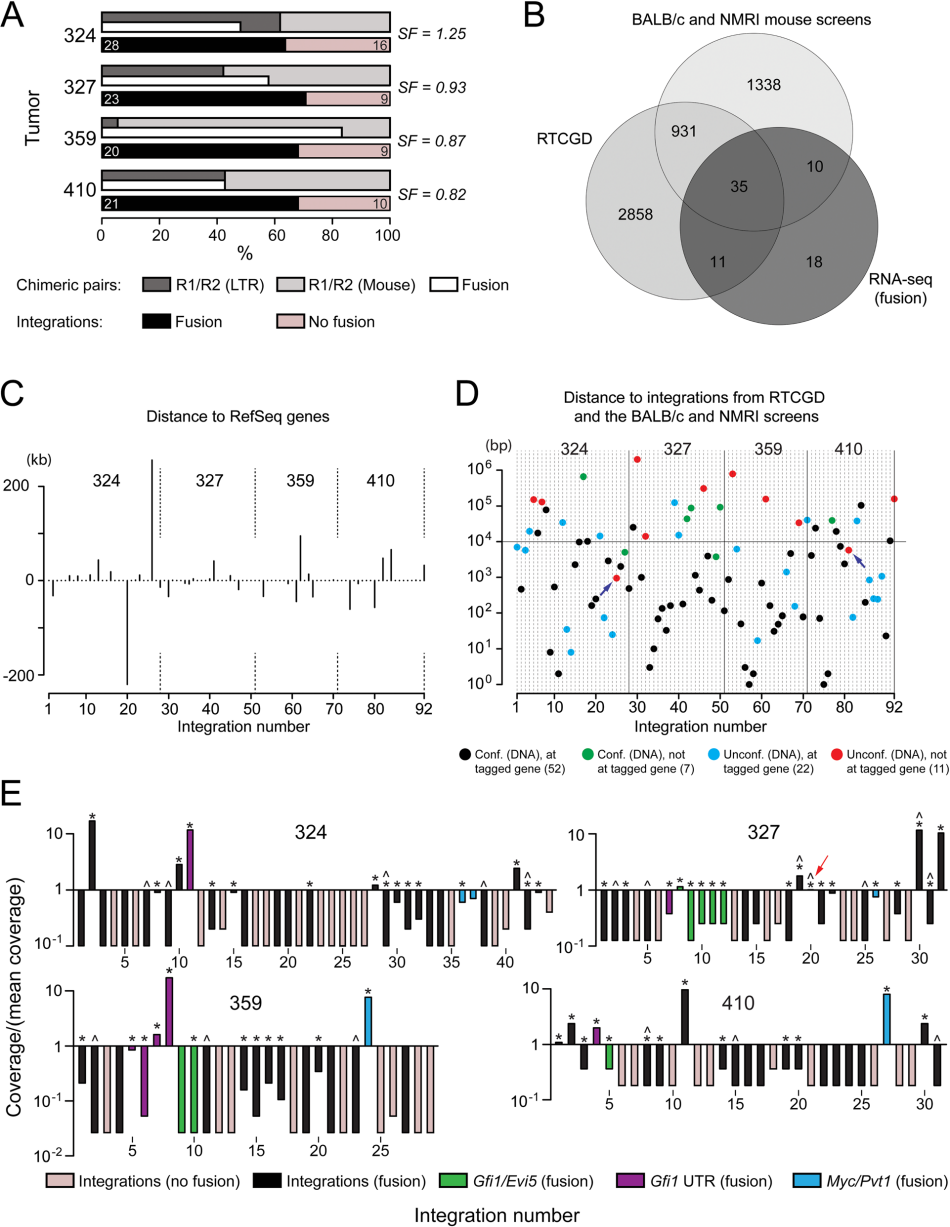
**Mapping, annotation and distribution of integrations from RNA-seq. (A)** Integration and library statistics for tumors 324 through 410 subjected to RNA-seq. Cufflinks [47] library scale factors (SF). The fraction of chimeric pairs (R1/R2) proceeding from the LTR or mouse sequence, respectively, as well as the fraction of pairs spanning proviral-mouse junctions (fusions) are shown. The percentage of integrations supported by fusions is shown in black while those without are shown in pink. Numbers within bars indicate the number of integrations. **(B)** Venn diagram showing the overlap (*P* = 2.03E-30*,* hypergeometric probability) of genes assigned from integrations containing a chimeric fusion with those assigned from integrations in RTCGD and the BALB/c and NMRI datasets in Table 1. **(C)** Distance map showing the positions of RNA-seq integrations supported by chimeric fusions relative to nearest RefSeq gene annotation. **(D)** Distance map showing the distribution of RNA-seq integrations relative to integrations from RTCGD and the BALB/c and NMRI datasets. The horizontal line marks a distance of 10 kb. This figure shows (per integration) gene assignments common to the integration datasets, and if integrations were confirmed by DNA analyses (the numbers in parenthesis indicate the number of integrations). Integrations marked by purple arrows may have been assigned to a different gene in **(B)** (described in the main text). **(E)** Coverage of each integration site relative to the mean coverage of all integrations in each tumor. The minimum coverage corresponds to a single chimeric read pair. The coverage of the integration marked by a red arrow in tumor 327 is above the mean. (*) integrations supported by chimeric fusions and confirmed in DNA analyses. (^) integrations supported by chimeric fusions that were not assigned to a previously tagged gene. The numbering of the integrations in C-E follows the numbering in Additional file 2.

In this study RNA-seq was used without specific enrichment to map integrations, and simultaneously determine transcript expression levels at sites of integration. We made whole-transcriptome libraries of four thymic tumors induced by wild-type SL3-3 in mice of an inbred NMRI strain. The four tumors are referred to as 324, 327, 359 and 410. We obtained ~290 million 101-base reads exceeding an average of ~70 million single reads per tumor (online available sequencing data). Integrations were mapped by analysis of 14 paired-end sequence signatures that expose genetic structural alterations in tumors (Additional file 1: Figure S1). We manually examined the evidence for each retroviral-mouse chimeric position and assigned 92 integrations supported by chimeric fusions, and 44 regions for which fusions were not directly covered in sequencing (Figure 1A). Table 1 provides an overview of the RNA-seq and other integration datasets used in this study. The complete lists of integrations from RNA-seq and the splinkerette-based PCR screen of NMRI mice in Table 1 are provided in Additional files 2 and 3, respectively.

**Table 1:** Retroviral integration datasets used in this study

**Integration dataset**	**Method**	**Model**	**No. integrations**	**Reference**
RNA-seq	RNA-seq	MLV SL3-3	136	Additional file 2
*BALB/c and NMRI	Splinkerette-PCR	MLV SL3-3	6117	[48-50]
**RTCGD	Mixed	Mixed	6749	[51]

(*) This dataset contains the combined number of unique integration coordinates from a previously published screen of BALB/c mice as well as the NMRI mouse screen provided in Additional file 3. (**) The Retrovirus and Transposon tagged Cancer Gene Database.

We observed a proportional increase in the number of integrations with increasing sequencing depth, and with the exception of tumor 359, the fraction of chimeric read pairs aligning in a direction from mouse to viral sequence, and vice versa, were nearly evenly distributed (Figure 1A). This indicates that both mouse and retroviral promoters contribute to chimeric transcription in tumors. In tumor 359, the nearly one-sided distribution of chimeric read pairs (from mouse to viral sequence) resulted from integrations into growth-factor independence 1 (*Gfi1*), where chimeric transcription is enhanced by high levels of mouse transcription (described below).

Comparison of RefSeq gene annotations showed significant overlap (*P* = 2.03E-30, hypergeometric probability) (Figure 1B) of genes assigned from integrations supported by chimeric fusions to those assigned from the conventional integration datasets in Table 1. Since these datasets comprise only 5,183 or 17% of RefSeq annotations gene assignment from the corresponding RNA-seq integrations is nonrandom. The distribution of the integrations with respect to the closest genes is shown in Figure 1C. Genes assigned from RNA-seq integrations that were not supported by chimeric fusions also showed significant overlap (*P* = 1.87e-08, hypergeometric probability) with genes assigned from the conventional screens. However, the potential risk of misassignment is higher since the exact locations of proviruses are not known (the annotations are available in Additional file 2).

We confirmed 59 of 92 (64%) integrations supported by chimeric fusions in DNA analyses using PCR (described in Methods). Since the majority of integrations that were not confirmed obtained a minimum coverage in sequencing (Figure 1E), it is possible that an unknown fraction did not amplify due to low copy numbers. In total, 22 out of 33 integrations that were not confirmed by DNA analyses could be assigned to a previously tagged gene (Figure 1D and E), while two out of the remaining 11 integrations were possibly misassigned in Figure 1B when considering their proximity to previous integrations (Figure 1D, purple arrows). Assuming that integrations which were neither confirmed by DNA analyses, nor assigned to a previously tagged gene are false-positives, the estimated error-rate is <12%. It should be noted that integrations supported by chimeric fusions showed a distribution at enhancer peak midpoints comparable to the BALB/c and NMRI integration datasets in Table 1 (described below).

We identified several integrations in RTCGD-assigned common integration sites (CISs) showing that multiple CISs are targeted in tumors: *Arf6*, *Ccnd3*, *Chd9*, *Coro1a*, *Frat1*, *Gfi1/Evi5*, *Hivep1*, *Hsp90b1*, *Ikzf1*, *Kis2*, *Mef2c*, *Mir17*, *Myb*, *Mycn*, *Pvt1*, *Rasgrp1* and *Thada* (Additional file 2). While the four tumors harbored integrations in *Gfi*1 and ecotropic viral-integration Site 5 (*Evi5*) (the *Gfi1/Evi5* locus), tumor 359 contained six integrations at this locus, four of which were positioned *in sense* in the 3′-UTR of *Gfi1* (Figure 1E). The expression of this gene was comparably high in tumors (sequencing data available online). The differential coverage at chimeric junctions in the *Gfi1* 3′-UTR in tumor 359 most likely represents the expansion of distinct cellular subpopulations. Tumor 327 contained six interspersed integrations at the *Gfi-1*/*Evi-5* locus, while tumor 410 contained two integrations at this locus. Tumor 324 contained only a single integration at the *Gfi1/Evi5* locus however it contained two integrations at the *Myc/Pvt1* locus. We have previously shown the occurrence of more than one integration at the same position in the same tumor, indicating that insertion at such positions trigger the onset of tumorigenesis and/or are facilitated by earlier stage or parallel mutations in individual leukemias [52] (discussed below).

### Novel principles of retroviral insertional mutagenesis

**Figure 2:**
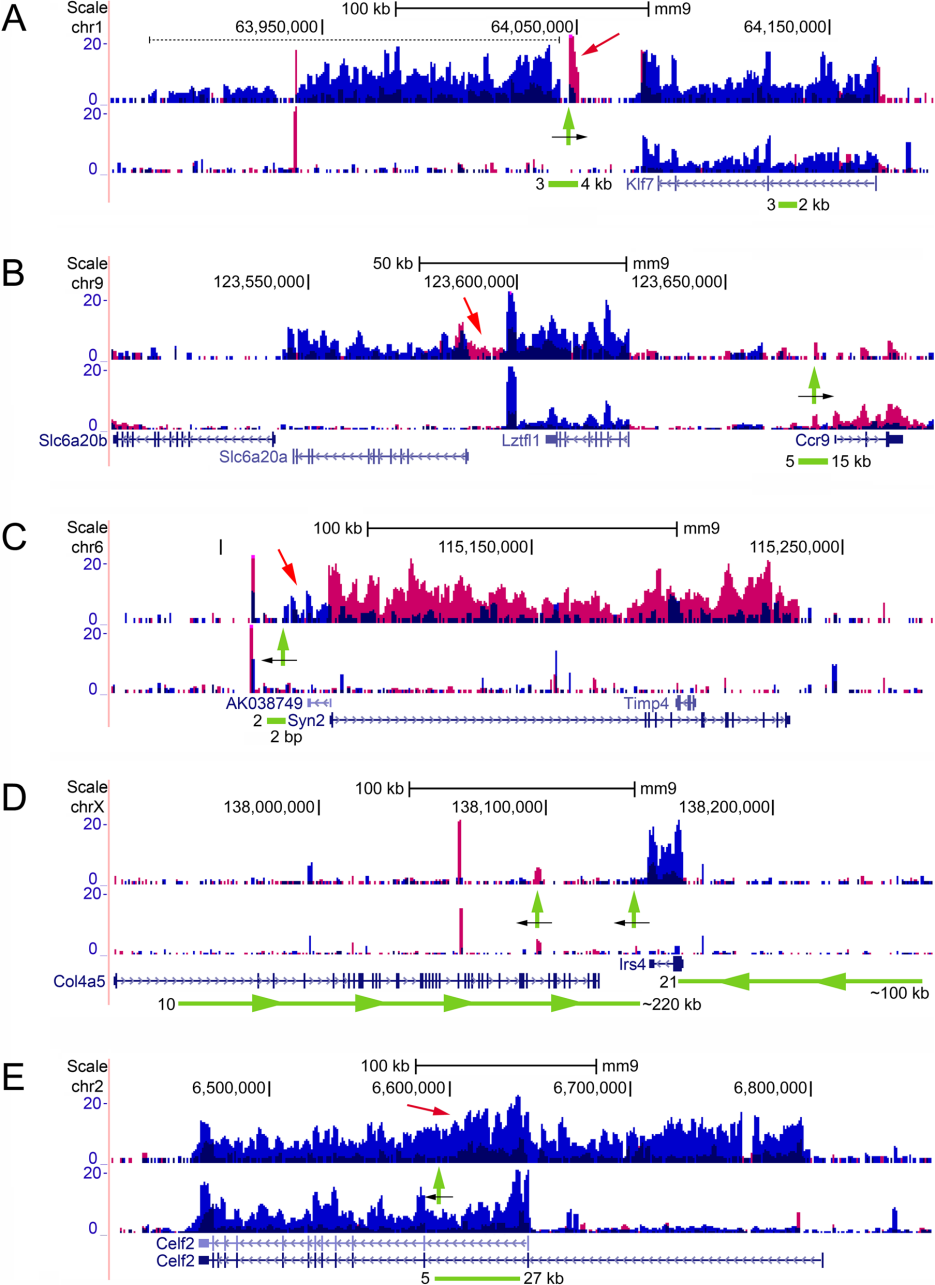
**Novel mechanisms of retrovirus-induced transcription activation in mouse tumors.** Deregulations are shown using normalized transcription coverage as BedGraphs [53]. Coverage on the plus and minus strand is colored red and blue, respectively. The upper panel in each subfigure **(A-E)** shows the transcription profile of a tumor containing a provirus. The lower panel shows the mean coverage of tumors without known integrations at the loci shown. Horizontal green lines mark integration clusters, and the number of proviruses from RTCGD and the BALB/c and NMRI datasets as well as the sizes of clusters are indicated. Vertical green arrows mark positions of proviruses identified in RNA-seq and orientations are indicated by black arrows. The deregulations in subfigures **A**, **C** and **E** are shown in detail in Figure 4. **(A)** Bidirectional activation. Integration induces upregulation of *Klf7*, activation of a large 165 kb unannotated region (dashed line), and local transcription activation at the provirus (red arrow). **(B)** Tandem-type activation. Integration at *Ccr9* activates *Lztfl1* and *Slc6a20a*, which are positioned adjacently on the DNA minus strand, as well as transcription of opposite polarity in the region in between these two genes (red arrow). **(C)** Sense/antisense activation. Integration downstream of *Syn2* activates transcription from both DNA strands resulting in expression of non-coding AK038749 (red arrow) and *Syn2*. **(D)** Activation of genes that are not targets of integration. *Col4a5* functions as a hotspot for retroviral integrations that activate expression of the distal gene *Irs4*, without affecting expression of *Col4a5* itself. Integration clusters marked with arrow heads indicate that proviruses share the same orientation. **(E)** Combination-type activation. Enhancer activation mutagenesis, promoter insertion and alternative splicing are used simultaneously by a single provirus to alter the expression pattern of *Celf2*. The red arrow marks increased transcriptional activity in the intron containing the provirus (described in the main text).

**Figure 3:**
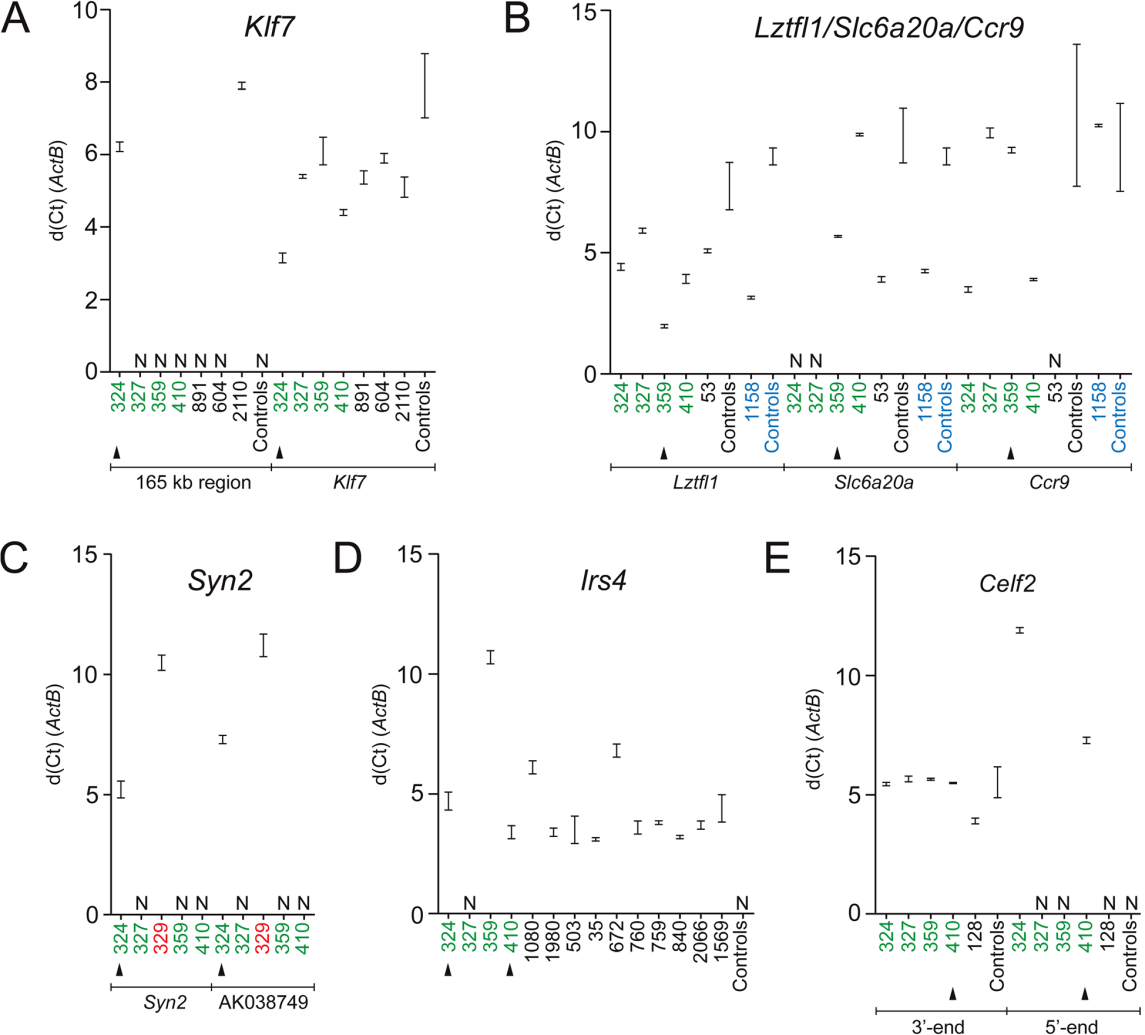
**The transcriptional deregulations are common to other tumors containing proviruses at the same positions. (A-E)** Reverse transcription (RT)-qPCR analyses including other tumors harboring integrations at deregulated loci. Tumors subjected to RNA-seq are colored green, and black arrow heads mark those that were found to contain a provirus at each of the loci. Other tumors of spleen and thymus from the NMRI dataset are shown in blue and red, respectively, while tumors from the BALB/c dataset are shown in black. As control, tumors from the same mouse strain and tissue were used without known integrations at the loci shown. It should be noted that the measurements for NMRI thymic reference tumors are shown individually, while the average of measurements is shown for NMRI and BALB/c splenic reference tumors, and these are marked as controls in the figure. (N) indicates that the Ct value was above a threshold of 30 cycles.

In the following sections we describe five novel mechanisms of IM that are shown in their genomic context in Figure 2. The activation mechanisms are distinguished by diverse patterns of mouse transcription deregulation including: bidirectional activation of mouse transcription on opposite sides of a provirus (Figure 2A); tandem-type activation of distal genes that are positioned adjacently on the same DNA strand (Figure 2B); sense/antisense-type activation of genes located on opposite strands (Figure 2C); activation of genes that are not direct targets of retroviral insertion (Figure 2D); combination-type IM where enhancer activation, alternative chimeric splicing and retroviral promoter insertion are used by a single provirus to alter the expression pattern of a mouse gene (Figure 2E). To confirm common activation patterns in other tumors harboring integrations at the same positions (Table 2) we used quantitative real-time PCR (qPCR) (Figure 3) and/or rapid amplification of cDNA ends (RACE) (described below). Fragments per kb of exon per million fragments mapped (FPKM) values for transcripts at the loci in Figure 2 as well as additional loci (described below) for individual tumors are shown in Additional file 4.

**Table 2:** **Integration sites at *Syn2*, *Klf7*, *Slc6a20a/Lztfl1/Ccr9*, *Celf2 *and *Col4a5/Irs4 *in tumors from BALB/c and NMRI mice**

**Tumor number and integration cluster**	**Integration site**
BALB/c	
35 (*Irs4*, upstream)	chrX:138171812
53 (*Ccr9*)	chr9:123667226
128 (*Celf2*)	chr2:6629104
503 (*Col4a5*)	chrX:138103923
604 (*Klf7*, downstream)	chr1:64047271
672 (*Irs4*, upstream)	chrX:138281270
759 (*Irs4*, upstream)	chrX:138295736
760 (*Irs4*, upstream)	chrX:138296394
840 (*Irs4*, upstream)	chrX:138294828
891 (*Klf7*)	chr1:64135021
1080 (*Col4a5*)	chrX:138066322
1569 (*Col4a5*)	chrX:138024869
1980 (*Col4a5*)	chrX:138079594
2066 (*Col4a5*)	chrX:137949312
2110 (*Klf7*, downstream)	chr1:64050480
NMRI	
329 (*Syn2*)	chr6:115073106
1158 (*Ccr9*)	chr9:123676544

This table lists integrations in other tumors from splinkerette-based BALB/c and NMRI mouse screens that were subjected to qPCR analysis and/or RACE. Integration clusters are shown in Figure 2.

### Bidirectional activation of *Klf7* and unknown RNA transcripts

**Figure 4:**
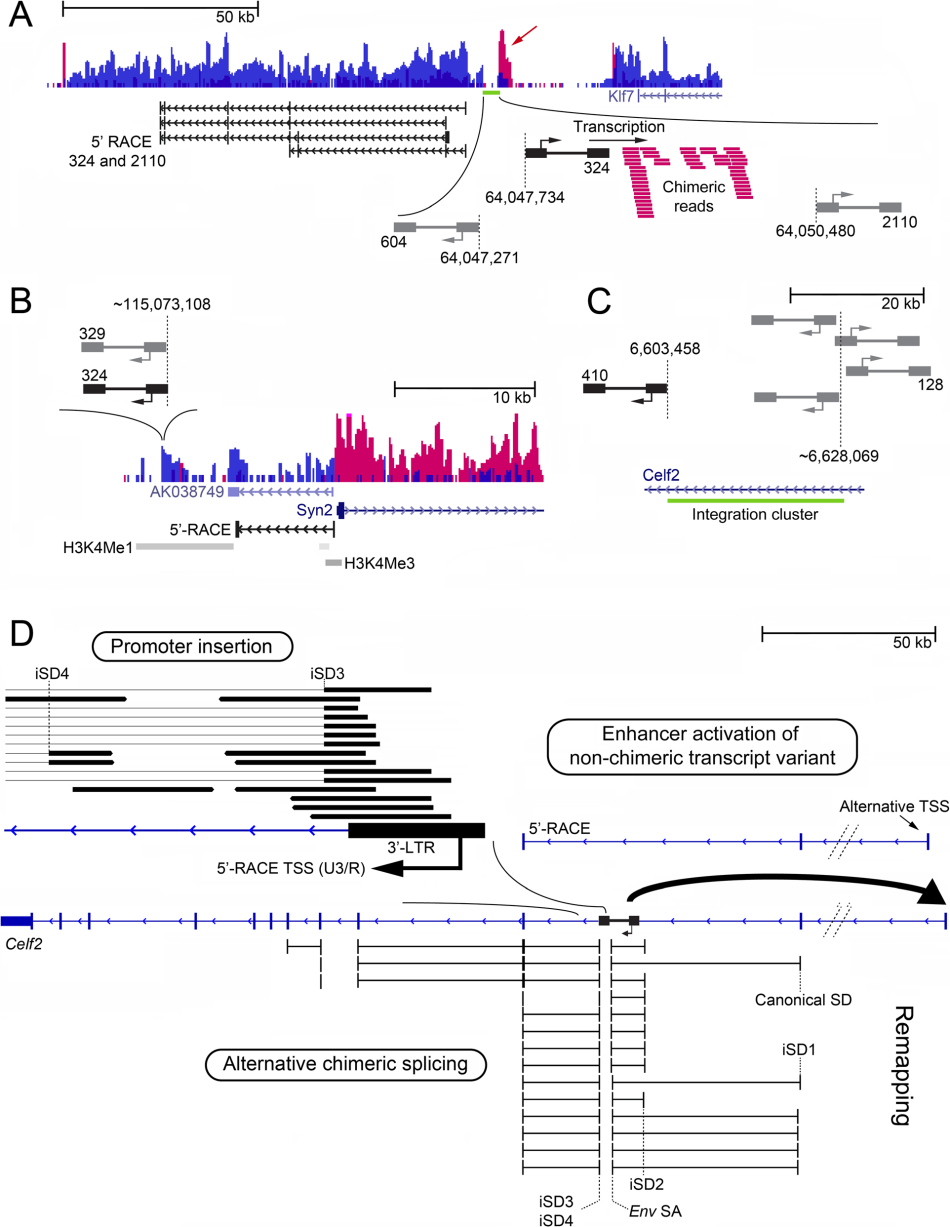
**Details of the bidirectional, sense/antisense and combination-type mechanisms at *Klf7*, *Syn2/*AK038749 and *Celf2*. (A)** Close-up of the *Klf7* downstream integration cluster. Three integrations are located in this cluster including one determined by RNA-seq in tumor 324 and two from the BALB/c mouse screen, one of which is positioned in the opposite orientation (604). RACE products obtained from tumors 324 and 2110 in the activated unannotated region are shown. At the 3′-LTR of the provirus in tumor 324 transcription proceeds in a direction from viral to mouse sequence (red arrow) showing that transcription is activated by promoter insertion at the integration site. **(B)** Close-up of the sense/antisense activation mechanism of AK038749 and *Syn2*. The proviruses are located in an H3K4Me1-enriched enhancer region in the *Syn2* EPU [25], located ~11 kb from the *Syn2* promoter (H3K4Me1/H3K4Me3-enriched region). The proximity (~200 bp) of *Syn2* and AK038749 suggest activation through a bidirectional promoter. AK038749 expression was also confirmed by RACE. **(C)** The *Celf2* intronic integration cluster contains five integrations in tumors from either RNA-seq (410) or the BALB/c mouse screen. In 410 the provirus is integrated ~25 kb from the other proviruses including the provirus in tumor 128 that was subjected to qPCR analysis (described in the main text). **(D)** The figure shows an alignment resulting from remapping of chimeric reads. Activation of *Celf2* involves a combination-type mechanism including promoter insertion, enhancer activation and alternative (chimeric) splicing. The non-chimeric upstream transcript variant, and initiation of transcription in the 3′-LTR, was confirmed by 5′-RACE. The stippled angled lines indicate shortening of the intron. The curved bold arrow illustrates retroviral enhancer activation. Intronic splice donor (iSD). Transcription start site (TSS). In **A-D**, the proviruses identified in RNA-seq are shown in black, and angled arrows denote the 5′-LTR. In **A-C**, tumor numbers are indicated and the approx. integration positions.

Integration at the *Klf7* locus induces a complex bidirectional-type activation pattern where major deregulation of mouse sequence is induced on both sides of the provirus (Figure 2A). This results in transcriptional activation of a large 165 kb region that is not annotated (dashed line), upregulation of *Klf7* as well as local activation of mouse sequence at the provirus (red arrow). The *Klf7* locus contains two integration clusters one of which is positioned ~35 kb downstream of *Klf7* while the other is located in the first intron of the gene. *Klf7* was found consistently overexpressed in tumors harboring proviral insertions (Figure 3A) however activation of the 165 kb region was restricted to two tumors (324 and 2110) that contained *in sense* integrations in the downstream cluster (Figure 4A). Therefore, activation of the 165 kb region appears to be dependent on the orientation of proviruses in the downstream cluster.

To determine whether the tumors shared common transcripts in the unannotated region, they were subjected to 5′-RACE which resulted in the identification of several unknown transcripts initiating at alternative start sites (Figure 4A). The longest transcript spanned ~90 kb within the activated region, while one transcript resulted from five splicing events. We did not identify any similarity hits in common gene or RNA databases (data not shown), suggesting that the transcripts are either random products or perhaps the remains of a gene that has lost sequence similarity to any known gene.

In principle, bidirectional activation could be thought to originate from the transcriptional activities of both LTRs of an intact provirus. However, based on RNA-seq chimeric transcription was only detected at the 3′-LTR/mouse junction, proceeding in a direction opposite to that of *Klf7* and the unannotated 165 kb region (Figure 4A). Therefore, transcription is induced locally by retroviral promoter insertion, while *Klf7* and the 165-kb region appear to be activated by enhancer activation mutagenesis.

Concurrent transcription from both LTRs was observed at other loci, including e.g. the *Tmem30b/Prkch* locus where antisense transcription (previously described by us [54]) and retroviral promoter insertion by the 5′-LTR and 3′-LTR, respectively, was detected by RNA-seq and 5′-RACE (Additional file 1: Figure S2). Integration at this locus also results in bidirectional mouse sequence activation, including transcriptional activation of unannotated sequence. Other loci for which unannotated transcripts were detected are described below.

### Integrations at *Ccr9* tandem-activate *Lztfl1* and *Slc6a20a*

We observed retrovirus-induced activation of independent transcripts at other loci including *Slc6a20a/Lztfl1/Ccr9* and *Syn2/*AK038749 (described below). Integration at *Ccr9* induces tandem-activation of the distal genes *Slc6a20a* and *Lztfl1* which are located adjacently in the same orientation (Figure 2B), as well as increased transcription of opposite polarity (red arrow). The *Slc6a20a*/*Lztfl1*/*Ccr9* locus contains five integrations clustered in a region immediately upstream of *Ccr9*. Therefore, based on closest distance, *Ccr9* would be expected to be a target of retroviral IM. However, we did not find this gene to be systematically deregulated in tumors suggesting that *Ccr9* is not activated by proviruses (Figure 3B). *SLC6A20* encodes an amino acid transporter, and is duplicated in mice, i.e. paralogues *Slc6a20a* and *Slc6a20b*[55]. *Slc6a20b* was not deregulated in any of the tumors harboring integrations at *Ccr9* (data not shown).

Therefore integration at *Ccr9* activates *Slc6a20a* and *Lztfl1*, exclusively, in three independent tumors analyzed. We speculate if the tandem-type activation pattern is induced by integration into a shared regulatory region through which proviruses can deregulate both *Slc6a20a* and *Lztfl1.* The regulation of genes through shared enhancers has been described previously and appears to be a regulatory principle that is applicable genome-wide [25,56,57].

### Sense/antisense activation of *Syn2* and non-coding AK038749

The sense/antisense activation mechanism at *Syn2/*AK038749 induces expression of the brain genes *Syn2* and non-coding AK038749 located in opposite orientation (Figures 2C and 3C). In human glioblastoma multiforme, *SYN2* and *TIMP4*, a metalloproteinase-encoding gene located within an intron of *SYN2*, are subjects to reciprocal deregulation [58]. We did not detect *Timp4* expression in any tumor by qPCR analysis using three different primer pairs (data not shown). We confirmed expression of AK038749 by 5′-RACE (Figure 4B). Interestingly, the integrations at *Syn2/*AK038749 are located in an enhancer region marked by the histone marker H3K4Me1 that is coregulated with the *Syn2* promoter, marked by H3K4Me3 and H3K4Me1, in mouse cerebellum and cortex [25] (Figure 4B). Since the AK038749 and *Syn2* transcription initiation sites are positioned only ~200 bp from each other, it is possible that proviruses activate a bidirectional mouse promoter. In fact, promoters of many coding genes transcribe non-coding RNAs in the opposite direction [59].

We have previously reported on the activation of neuronal neurogranin (*Nrgn*) in T-cell lymphomas induced by SL3-3 integration in the *Esam/Vsig*2*/Nrgn/Siae/Spa*17 locus where the *Nrgn* expression level in lymphoid tumors corresponded to brain levels [60]. Therefore, the deregulation pattern suggests that enhancer-promoter associations in mouse lymphomas, which are normally active in brain, are reinstated following retroviral integration, resulting in activation of oppositely located brain genes at this locus.

### Integrations at the *Col4a5/Irs4* locus activate *Irs4* only

The activation pattern at the *Col4a5/Irs4* locus shows resemblance to deregulations detected at the *Ccr9* locus. However, in this case one gene (*Col4a5*), functions as a hotspot for retroviral integrations that induce expression of another distal gene (*Irs4*) (Figure 2D), without affecting the expression of *Col4a5* itself in any tumor analyzed (Figure 3D, and data not shown). The *Col4a5/Irs4* locus contains in the excess of 30 integrations delimited by two major clusters that intersect at *Irs4. Col4a5* contains 10 of these sites, one of which is located more than 220 kb from the *Irs4* promoter. The finding that *IRS4* but not *COL4A5* activation in T-ALL involves translocation of both genes to the T-cell receptor beta locus [61] is particularly interesting considering that insertion of the retroviral enhancer mimics such oncogenic rearrangements. We detected an activation pattern at the *Wwox* locus fairly similar to the patterns observed at the *Klf7*, *Col4a5/Irs4* and *Ccr9* loci (Additional file 1: Figure S3). Integration into *Wwox* induces increased expression of *Wwox* as well as activation of distal *Maf* and unannotated mouse sequence of opposite polarity both of which are located outside the actual integration target.

### *Celf2* is activated by a combination-type mechanism of insertional mutagenesis

The deregulation shown in Figure 2E results from a combination-type activation mechanism where several modes of IM are employed by a provirus to alter the expression of *Celf2*, whereby a non-chimeric transcript variant initiating from a far upstream position is activated, including also increased transcription in the provirus-containing intron (red arrow).

Remapping of chimeric sequencing reads against the *Celf2* reference sequence containing the integrated provirus made it possible to link upstream and downstream *Celf2* sequence to the provirus (Figure 4D). RNA splicing is mediated by the viral *env* splice acceptor site, and involves three splice donor (SD) sites: the canonical SD located ~39 kb upstream from the insertion site; intronic iSD1 and iSD2, located ~38 and ~2 kb from the integration site, respectively. We confirmed splicing between the canonical SD site and the *env* splice site by RT-PCR and Sanger sequencing (data not shown). In the 3′-end of *Celf2*, splicing is mediated by iSD3 and iSD4, located 15 bp and 180 bp from the integration site, respectively. The coverage by sequencing reads at the 3′-LTR/mouse junction indicated that *Celf2* is also activated by retroviral promoter insertion. Transcription initiation at the canonical U3/R position was confirmed by 5′- RACE as shown in Figure 4D.

We wanted to confirm the activation pattern in other tumors containing proviruses in the *Celf2* integration cluster (Figure 4C). Due to inadequate tumor tissue the analysis included one tumor (128) in which the provirus is integrated in the opposite orientation. In this tumor only the 3′-end transcript levels were increased, showing that no transcript variant is expressed (Figure 3E). The implications of alternatively spliced variants of the CELF/Bruno-like family members is not fully understood, however differential expression of *Celf2* isoforms has been related to separate tissues, as well as fetal versus adult developmental stages [62]. Since, the tumors originate from similarly-aged adult mice (described in Methods) the non-chimeric variant initiating at a far upstream position in 410 is most likely induced by retroviral enhancer-activation and not a developmentally regulated difference. The lack of 5′-end activation in tumor 128 is possibly attributable to mouse strain or tissue differences and/or to the different position and orientation of the provirus in this tumor.

### Long-range IM is sustained by integration into enhancers in end-stage tumors

Gamma-retroviral vectors show a strong propensity for insertion into nucleosomal DNA, and in this study the integration-enrichment at enhancers of promoter-distal integration clusters was examined to determine epigenetic features underlying possible long-range gene activation in end-stage tumors. We exploited publically available ENCODE ChIP-seq data from Ren’s laboratory [25] (Additional file 1: Figure S4) and major splinkerette-based PCR integration datasets derived from tumors of more than 2,000 BALB/c and NMRI mice (Table 1). To account for possible bias introduced by genomic regions containing high numbers of integrations a supplementary reduced dataset was also analyzed which excluded highly tagged regions (described in Methods).

We found that the integrations were distributed in clusters peaking at immediate (~1,000 bp), intermediate (~8 kb) and distal (>10 kb) positions relative to UCSC TSSs (Table 3 and Additional file 1: Figure S5). Thus the majority of proviruses are not located in the immediate vicinity of TSSs. The enhancer-colocalization analyses are summarized in Table 3. The far majority of integrations colocalize with regions enriched in H3K4Me1 and/or H3K27Ac irrespective of their distance to TSSs, while the fraction of integrations colocalizing with the promoter marker H3K4Me3 decreases as expected.

**Table 3:** Wild-type MLVs show a strong propensity for insertion into enhancers irrespective of their distance to UCSC TSSs

	**TSS <3 kb (immediate)**	**3-10 kb (intermediate)**	**Beyond 10 kb (distal)**
Complete (n = 6117)	1602 (26%)	1186 (29%)	3329 (54%)
Median distance (bp)	868	5872	33169
H3K4Me1/H3K27Ac	1494 (93%)	1062 (90%)	2757 (83%)
H3K4Me3	902 (56%)	164 (14%)	479 (14%)
Reduced (n = 2127)	604 (28%)	348 (16%)	1175 (55%)
Median distance (bp)	800	5924	37532
H3K4Me1/H3K27Ac	551 (91%)	272 (78%)	803 (68%)
H3K4Me3	438 (73%)	59 (17%)	137 (12%)

Summary of the colocalization-analyses between retroviral integrations in tumors and ChIP-seq enhancer (H3K4Me1 and H3K27Ac) markers. The far majority of integrations are located at intermediate and distal positions relative to TSSs. The median distance to TSSs of integrations in each cluster is shown, as well as the total number of integrations that colocalized with ChIP-seq markers from thymus and spleen, combined. Statistics concerning the H3K4Me3 marker shows integrations that also colocalized with enhancer markers.

**Figure 5:**
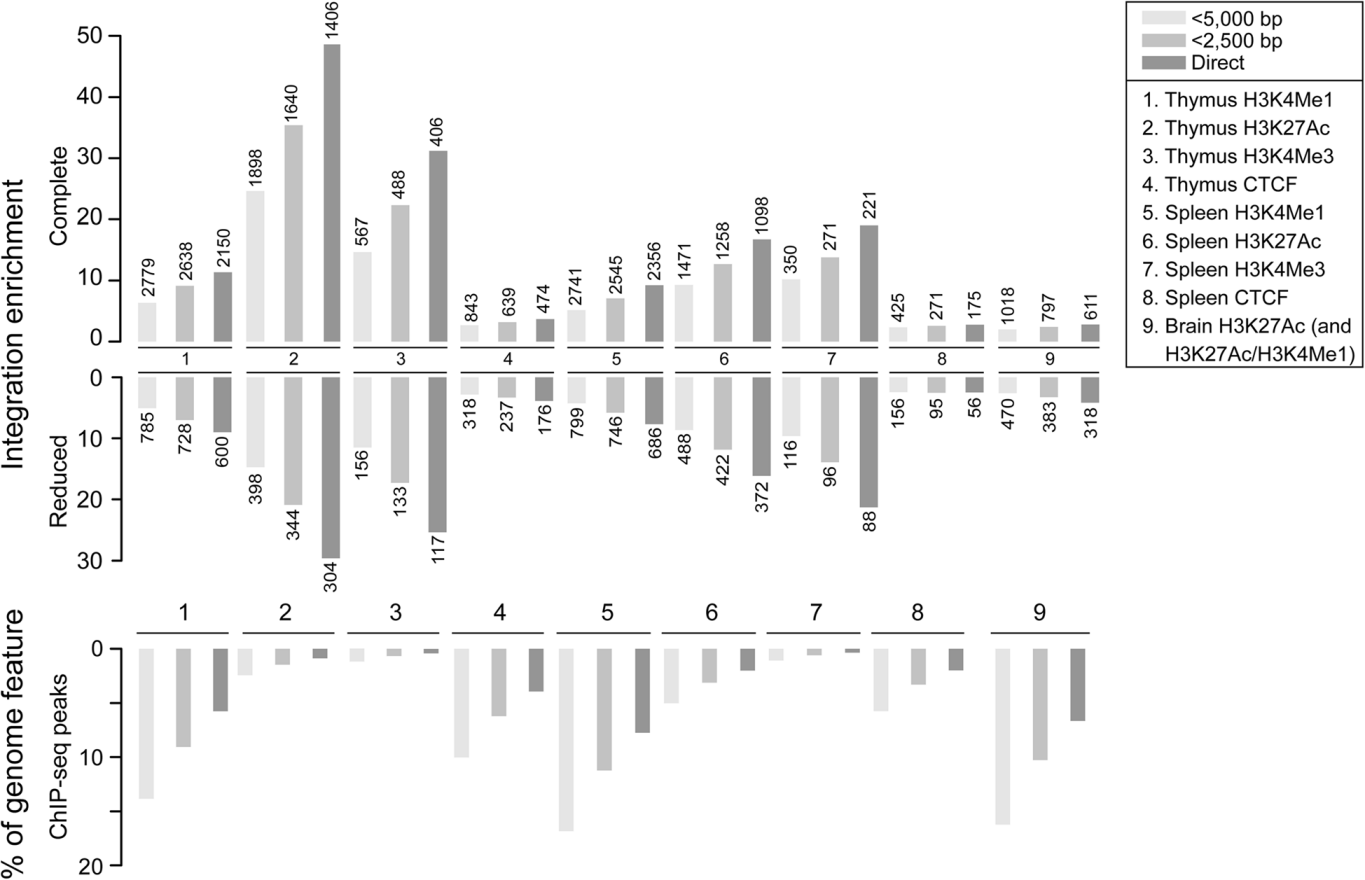
**Integrations are highly enriched at enhancers.** Integration-enrichment analyses of promoter-distal integration sites with enhancers from thymus, spleen and brain including also the insulator CTCF and promoter marker H3K4Me3. The number associated with each bar indicates the absolute number of integrations that colocalized with a given ChIP-seq marker. We defined direct colocalization based on extension of ChIP-seq peaks by 1,250 bp (described in Methods). The enrichments shown were highly significant compared to random (*P* < 0.001). The lower panel shows the percentage of the genome that the ChIP-seq features comprise including sequence extensions up to 5,000 bp.

We determined integration-enrichments of promoter-distal integrations at ChIP-seq peaks (Figure 5), and observed strong enrichments at H3K4Me1, H3K27Ac and H3K4Me3 regions of lymphoid tissues compared to random (*P* < 0.001 for all enrichments shown, described in Methods). The chromosomal distribution of integrations colocalizing directly in Figure 5 is shown in Additional file 1: Figure S6. At a distance of 5,000 bp from the juncture of ChIP-seq peaks the sequential decrease in colocalizing integrations was nearly proportional to random for markers including H3K4Me1 and the insulator CTCF (data not shown), as a result of the larger amount of the genome these markers comprise when extended to 5,000 bp (Figure 5). Considering direct overlaps, the integration enrichments reached as high as ~50 and ~30-fold (H3K27Ac) for the complete and reduced integration sets, respectively, showing that proviruses target enhancers directly. In total 2,757 of 3,329 (83%) promoter-distal integrations were positioned at enhancer markers of the lymphoid tissues, while the number for the reduced dataset was 803 of 1,175 (68%) (Table 3). The distribution of promoter-distal integrations relative to specific ChIP-seq enhancer markers is shown in Additional file 1: Figure S7.

**Figure 6:**
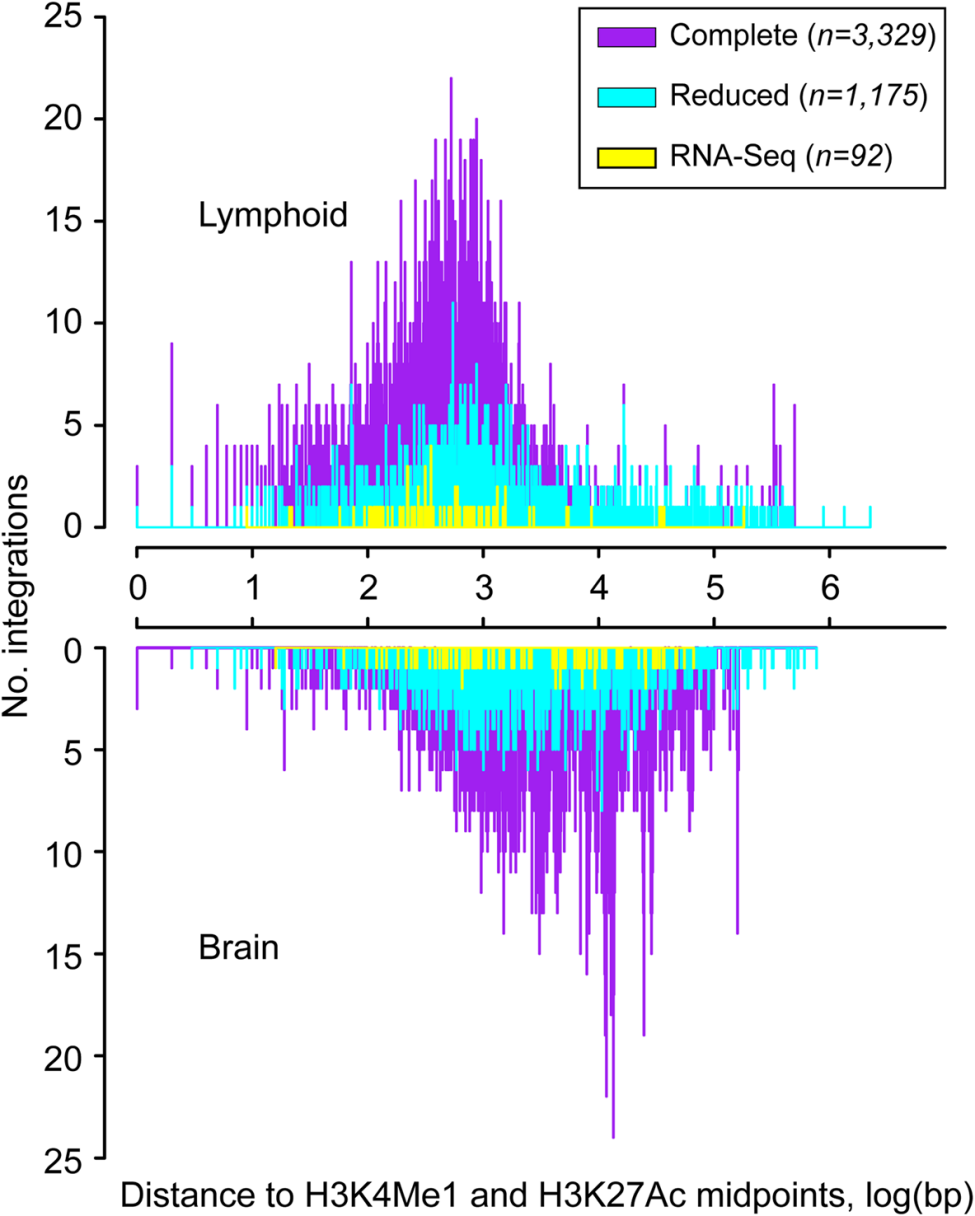
**The colocalization of integrations with enhancers is tissue-specific.** The figure shows the distribution of promoter-distal integrations relative to enhancer midpoints determined from lymphoid (i.e. spleen and thymus) H3K4Me1 and H3K27Ac ChIP-seq peaks. The results from H3K4Me1 and H3K27Ac control datasets from brain (i.e. cerebellum and cortex) shows a substantial distortion in the distribution of integrations relative to enhancer midpoints. The RNA-seq integrations supported by chimeric fusions show a tissue-specific distribution that is comparable to the larger integration sets.

We observed consistently lowered enrichment of integrations at enhancers of brain indicating that retroviral integration targets enhancers tissue-specifically, and therefore the enrichments in most cases reflect integration into enhancers that are common to brain and the lymphoid tissues (data not shown). The distribution of promoter-distal integrations with respect to the midpoints of H3K4Me1 and H3K27Ac ChIP-seq peaks shows that the proviruses form a dense cluster peaking at ~800 bp in case of lymphoid enhancers (i.e. spleen and thymus), while the distribution is markedly distorted in case of brain enhancers (i.e. cortex and cerebellum) (Figure 6).

Considering overlapping integrations in Table 3 at intermediate and distal positions, ~12-17% of the integrations overlap both H3K4Me1/H3K27Ac and H3K4Me3 markers. Enrichment of both enhancer and promoter markers may be caused by physical interaction between these regulatory elements as predicted by DNA looping, whereby the histone modification is assigned to the interacting region [63-65]. It could also represent regions at unannotated TSSs, where proviruses are located at promoters and promoter-proximal enhancers resulting in a colocalization pattern similar to the promoter-immediate integrations in Table 3.

## Discussion

In this study we used RNA-seq and ENCODE ChIP-seq data to determine positions of integration, local patterns of mouse sequence deregulation and integration-enhancer colocalization in end-stage tumors induced by the SL3-3 retrovirus. We mapped 136 integrations in only four tumors. 59 out of 92 (64%) integrations supported by chimeric fusions were confirmed by DNA analyses (Figure 1D and E), while the majority of these (~90%) showed an expected distribution close to enhancer midpoints (Figure 6). While the number of integrations in only four tumors may seem high comparably high numbers of integrations in transposon and retroviral tumor models have been reported previously [45,66]: e.g. deep sequencing analysis of mouse mammary tumor virus (MMTV)-induced tumors, revealed an average of 27 integrations per tumor, a number that was significantly higher when including integrations supported by only a single chimeric sequencing read [45].

We cannot completely exclude that some insertions that had low coverage in RNA-seq may represent endogenous false-positives even-though they were unique for each tumor. We anticipate that factors such as viral mutation and/or recombination during tumorigenesis may complicate validation of genuine integration events, and would therefore require optimization of PCR conditions and cloning of bands that differ from an expected size. Mutant viruses were indeed detected in this study by Sanger sequencing including several 72-bp repeat variants of SL3-3 which are known to accumulate in end-stage tumors [67,68] (data not shown). MLVs also frequently recombine with endogenous sequences to generate replication-competent derivative viruses which add to the complexity of MLV-induced tumors [69,70].

The majority of integrations could not be correlated with local transcriptional activation by Cufflinks differential expression analysis (online available sequencing data) using known gene annotations though infrequent activation of mouse (unannotated) sequence was observed at several positions as described throughout this manuscript. We have previously shown that integration into major CISs, including *Myc* and *Rasgrp1*, does not consistently induce measureable changes in transcript levels, indicating that deregulations may be imposed post-transcriptionally, in some cases [52]. This could happen following integration within a gene where premature polyadenylation induced by the retroviral poly (A) signal results in a truncated transcript and a protein with possibly altered function rather than increased gene expression, reviewed in [4,5]. In addition, searching for disrupted target regions is also complicated by the fact that deregulations may be imposed over long distances. Alternatively, subpopulations of cells that constitute a minor fraction of clonal tumors may become out-averaged in RNA-seq, thereby masking events of retrovirus-induced transcription activation.

We found multiple integrations in the same loci, and in the same tumors, including *Gfi-1*/*Evi-5* and *Myc/Pvt1*. The probability of finding multiple integrations in the same region in one tumor by chance is exceedingly small. Therefore, integrations into such positions are either pivotal for tumorigenesis or promoted in a context-dependent manner. An example is *LMO2* and *IL2RG* cooperation in leukemia cases among X-SCID patients where insertional mutagenesis of *LMO2* results in an increased growth advantage in the presence of *IL2RG*[71,72]. Therefore, earlier stage or parallel mutations may predispose progenitor cells for gaining an even further growth advantage following insertion into certain loci. Integration into CISs is commonly considered important as such integrations should mark disruptions that triggered the onset of tumorigenesis in progenitor cells [73], and in this study numerous integrations in RTCGD-assigned CISs were detected e.g. *Ccnd3*, *Frat1*, *Gfi1/Evi5*, *Myb* and *Myc/Pvt1* (Additional file 2). Cancer genomes frequently acquire mutations that alter disease progression and become subject to purifying selection in late stages of tumorigenesis [74-76] suggesting that integrations in subsidiary loci may contribute to progression rather than onset.

The human orthologs of the genes described in this study are implicated in cancer (Figure 2 and Additional file 1: Figures S2-S3 which show deregulations of the *Prkch* and *Wwox* loci, respectively). Elevated levels of the transcription factor *KLF7* is associated with minimal residual disease and relapse following chemotherapeutic treatment of childhood acute lymphoblastic leukemia (ALL) and *KLF7* also promote early T cell survival [77,78]. *SYN2* encodes a neuronal phosphoprotein (Synapsin 2) and is deregulated in human glioblastoma multiforme and breast cancer [58,79]. As described already, *IRS4* is activated in T-ALL following *COL4A5/IRS4* translocation involving the T-cell receptor beta locus [61]. The CUGBP-ETR3-like factors and Bruno-like (or CELF/Bruno-like) family of RNA-binding proteins regulate RNA splicing, translation and mRNA stability. The family comprises six members including *CELF2*, which functions as a tumor suppressor in colon cancer [80]. *TMEM30B* and *PRKCH* which are located adjacently on human chromosome 14 have been implicated in brain and breast cancer, respectively [81,82]. *SLC6A20*, *LZTFL1* and *CCR9* are located on 3p21.3 in a region that is often found eliminated in tumors, referred to as CER1 (commonly eliminated region 1). Chromosome 3 abnormalities have been proposed to mediate tumor formation due to loss of putative tumor suppressor genes [83-85]. *WWOX* also functions as a tumor suppressor in cervical cancer where it induces apoptosis and inhibits proliferation [86]. Although such insertional activation of tumor suppressors appears at least multifaceted, tumorigenesis may be augmented by overexpression of tumor suppressors which themselves may also exhibit oncogene properties context-dependently [87,88].

It has become widely established that retroviral vector integration favors nucleosomal over naked DNA, including the epigenetic promoter and enhancer markers H3K4Me3 and H3K4Me/H3K27Ac, respectively [18-24]. Importantly, the studies by De Rijck *et al*. and Sharma *et al*. have exposed a fundamental mechanism for integration of MLVs at TSSs mediated by BET proteins [29,30]. Therefore, BET proteins appear to form the MLV counterpart of HIV LEDGF/p75. These studies are particularly interesting with respect to understanding fundamental integration site selection patterns, as well as the underlying chromatin structure that promotes host transcription activation by an integrated retrovirus. While the majority of MLVs do not target TSSs it is currently not known whether BET proteins perform a universal function in directing proviruses to distal regions.

Short-term models provide limited information, if any, on the selection pressure during tumorigenesis, and in our study integration-enhancer colocalizations in end-stage tumors from more than 2,000 mice was examined. We found that ~45% of the integrations were concentrated in clusters at immediate and intermediate distances from TSSs, while the remaining integrations were dispersed throughout an extended promoter-distal cluster (Table 3 and Additional file 1: Figure S5). We found that proviruses located promoter-distally showed a strong propensity for integration at enhancers (83%) comparable to integrations positioned in closer vicinity to TSSs (~90%), and this tendency was also confirmed in a reduced integration dataset where colocalization is not biased by highly tagged genomic regions (Figure 5 and Table 3). Considering that ~50% of ENCODE H3K4Me1 and H3K27Ac ChIP-seq sequence of spleen and thymus (together comprising ~181 Mb in total) are located promoter distally [25] (i.e. beyond 10 kb from UCSC TSSs [89]), the proportion of colocalizing integrations in promoter-distal regions, as well as the dense clustering of integrations at enhancer midpoints (Figure 6) accentuates that enhancers are indeed major targets of MLV integration in tumors. Our results are comparable to the studies by De Ravin *et al*. [24] and LaFave *et al*. [22], where it was found that ~87% of vector integrations in CD34+ cells overlap H3K4Me1-enriched ChIP-seq peaks throughout the genome [24], and that integration is driven by promoters and strong enhancers in human HepG2 and K562 cells [22].

We found that a considerable fraction of proviruses did not directly target enhancers rather they appeared positionally offset or scattered randomly in the genome (Figures 5 and 6). For the reduced dataset offset integrations accounted for 32% of all promoter-distal integrations, and 22% in case of intermediately positioned proviruses (Table 3). The integration pattern is likely influenced by the chromatin state of individual end-stage tumors [90,91], and therefore it is difficult to estimate an absolute fraction of integrations that target enhancers which optimally requires the sequencing of hundreds of tumors in this particular case. Considering the looping model in which a promoter-distal enhancer is brought into proximity of a promoter, perhaps positional offsets or displacements of retroviral integrations reflect local structural features of the genome whereby the LTR becomes favorably positioned for interaction with a host promoter. Taking into account the size of a provirus or a retroviral vector, it seems plausible that some degree of local structural reorganization of the genome should accompany integration. In the study by Zhang *et al*. [35] the authors observed positional expansion, comprising several kb, of enhancers (H3K4Me1) in tumor cells containing proviruses at the *c-Myb* locus. It would be interesting to address at a large-scale how the local chromatin environment changes in response to retroviral integration in tumors, as well as determine BET protein binding sites genome-wide. This should show if BET tethering comprises a mechanism for integration outside TSSs, and possibly also account for integrations offset from enhancers.

In its basic form, a retroviral vector contains a therapeutic gene in place of the *gag*, *pol*, and *env* genes, and is delivered in the form of a replication-defective virus particle. Although vector-based treatments have proven highly effective in human clinical trials IM constitutes a major safety concern due to the development of leukemias in a minority of patients following treatment of SCID and CGD [10-15] (described above). In this study we have exposed several novel principles of gamma-retroviral-induced deregulations which altogether share a prominent complexity that reaches beyond any previously described disruption (Figure 2 and Additional file 1: Figures S2-S3).

We have shown several examples where more than a single gene is subject to activation from the same or opposite DNA strands including both proximal genes and genes whose initiation sites are positioned distantly from each other (the AK038749/*Syn2* and *Slc6a20a*/*Lztfl1/Ccr9* loci). We have shown that unannotated RNAs are transcribed at sites of integration as well as in more distal regions (the *Klf7*, *Tmem30b/Prkch* and *Wwox* loci), and that the provirus may activate transcription outside the actual gene in which it is integrated (the *Col4a5/Irs4* and *Wwox loci*). We have also shown occurrences of combination-type activation patterns where retroviral promoter insertion and enhancer activation mutagenesis are employed by a provirus to alter the expression of a single gene (*Celf2*) as well as genomic regions outside genes (the *Klf7* and *Tmem30b/Prkch* loci).

It may seem counterintuitive that retrovirus-induced deregulations should in general be restricted to a single mode of mutagenesis however there is a remarkable absence of studies reporting the use of combination-type activation mechanisms. One reason could be that it is difficult without prior knowledge to dissect deregulations involving chimeric and non-chimeric transcripts, respectively, to the overall expression pattern at a target locus considering also (1) transcription from the non-infected allele, and (2) unannotated transcripts. We believe that combination-type deregulation may represent a more general mechanism exposed by whole-transcriptome RNA-seq in our study.

The promoters of many coding genes transcribe non-coding RNAs in the opposite direction, and are therefore bidirectional *per se*[92], and in cancers, non-coding RNAs specific for certain malignant phenotype as well as pseudogenes are commonly expressed [93,94]. Moreover, regulation through enhancer-promoter interaction is not strictly pairwise, rather enhancers may be shared between separate promoters to intricately coregulate the expression of more than one gene [25,56]. Enhancers may contribute to the establishment of a intra and interchromosomal three-dimensional regulatory networks [95], and they may also be positioned in genes located adjacently to the genes that they regulate [96]. Noncoding intergenic transcription may regulate nearby protein-coding genes and an L1 LINE at *AZU1* provides an example where expression of this non-LTR retrotransposon correlates with the expression of multiple surrounding genes in addition to *AZU1*[97]. Considering the human globin locus, an ERV-9 LTR-element modulates long-range transcription factor occupancies at multiple *cis*-linked genes thereby coordinating gene switching during hematopoiesis, and it also activates intergenic RNAs at low levels as a result of transient DNA looping with multiple intergenic sites [98]. Therefore, at least for retrotransposons transcriptional activities are sustained which present a complexity comparable to the transcription patterns in MLV-induced tumors.

Taken together the deregulations described by us comply with emerging principles of complex higher-order genome regulation and show that MLVs have evolved to hijack such routes to activate multiple regions resulting in complex and long-ranging deregulations that are difficult to evaluate using conventional methods. Our analyses of integration-enhancer colocalizations in tumors also strongly support such models. Therefore, we anticipate that retrovirus-induced deregulations of equivalent or similar complexity are broadly applicable to other loci throughout the genome.

## Conclusion

The analysis of MLV-induced mouse tumors using RNA-seq has revealed novel mechanisms of retroviral insertional mutagenesis resulting in deregulations that reach beyond a single and nearest annotated gene target. Awareness of this previously undescribed layer of complexity regarding host sequence activations may prove important for elucidating adverse effects in retroviral-based gene therapies. We have also shown that wild-type gamma-retroviruses are positioned at enhancers of lymphoid tumors irrespective of their distance to TSSs, showing that insertion into regulatory regions is highly specific and also subject to positive selection during tumorigenesis. This suggests a mechanism whereby the provirus exploits the higher-order genome regulatome for sustaining long-range deregulations in tumors. This study should prove useful for extrapolating adverse outcomes of retroviral vector therapies, and for understanding fundamental cellular regulatory principles.

## Availability of supporting data

Sequences are available from the NCBI short read archive (accession no. SRP041565). Primer sequences not provided in Additional file 5 are available on request.

## Methods

### Mouse infection and splinkerette-based PCR mapping of integrations

Inbred BALB/c and NMRI mice were infected with the rapid lymphomagenic MLV SL3-3 strain as described previously [48-50]. Upon disease or appearance of tumors (in 60 to 70 days) the mice were sacrificed and spleen and thymus organs eviscerated and kept frozen at −80°C. The approx. size of thymic tumors was ~1.5 cm in the longest dimension while that of splenic tumors was ~3 cm. DNA was extracted using the DNeasy Tissue kit (Qiagen) and integrations determined using an automated high-throughput splinkerette-PCR method [36,38]. We have previously published results from the screen of ~2,000 BALB/c mice (Table 1). The NMRI dataset in Additional file 3 contains integrations from several cohorts of mice infected with SL3-3. The total number of mice was 120, including a cohort of 22 mice from which the four tumors subjected to RNA-seq originate.

### RNA isolation

Total RNA was extracted from thymus and spleen by the guanidinium thiocyanate-phenol-chloroform method (TRIzol, Invitrogen) and purified on spin columns using the RNeasy Mini Kit (Qiagen), including on-column DNase I treatment (Qiagen), and stored at −80°C. The concentration and purity was routinely determined on a NanoDrop (Thermo Scientific), and RNA integrity was assessed by microfluidics technology on the Agilent BioAnalyzer 2100 (Agilent).

### cDNA library preparation and sequencing

Total RNA from four SL3-3-induced NMRI mouse tumors was depleted of rRNA by hybridization using Ribo-Zero rRNA Removal Kit for Human/Mouse/Rat (Epicentre). The samples were then concentrated with RNA Clean & Concentrator (Zymo Research), and cDNA libraries prepared with the ScriptSeq RNA-Seq Library Preparation Kit (Epicentre) following the manufacturer’s guidelines. The libraries incorporated barcodes for multiplexed sequencing using the RNA-Seq Barcode Primers for Illumina (Epicentre). The resulting cDNA libraries were amplified by limited 12-cycle PCR and size-fractionated using high-percentage agarose gel electrophoresis. The size of the sequenced libraries was 400–450 bp including adaptors. The libraries were selectively quantified on a LightCycler 480 II Real-Time PCR System (Roche), using the KAPA Library Quantification Assay (Kapa Biosystems). The libraries were pooled in equal amounts on one paired-end flow cell lane using the cBot cluster generation process (Illumina), and sequenced on an Illumina HiSeq 2000 producing 2x101-base sequencing reads.

### RNA-seq-based integration mapping

To filter chimeric read pairs a custom reference was built manually using BLAT [99] based on the content of SL3-3 LTR-like sequence in the NCBIM37/mm9 mouse reference assembly (Additional file 1: Figure S8). In brief, SL3-3 LTR [GenBank:AF169256] sequence windows were aligned to the mouse genome in 5 bp increments to identify mouse sequence windows showing 90% or higher identity by sequence. The custom reference included also SL3-3 sequences [GenBank:X00862 and GenBank:AF169256]. Subsequently, 25 bp sequencing reads were mapped against the custom reference with the Burrows-Wheeler Aligner (BWA) [100] followed by sequestration of read pairs in which only one of the mates mapped. To identify integrations full length reads were quality trimmed and aligned separately with BLAT against the NCBIM37/mm9 reference assembly modified to contain SL3-3 on a separate chromosome. The chimeric pairs produce either one of 14 paired-end sequence signatures, based on U3, U5, SD or no chimeric fusion coverage (Additional file 1: Figure S1). We manually examined the evidence for each of the integrations and integrations common to the samples were considered endogenous false-positives based on the improbability of insertion at the exact same position in several samples. Integrations supported by a chimeric fusion contained intact sequence, at one or both LTRs. In any case the minimum requirement was 40 bp of unambiguously mapped sequence. The integrations are listed in Additional file 2.

### Analysis of ENCODE data

ChIP-seq data [25] were obtained from ENCODE (Additional file 1: Figure S4), while SL3-3 integrations were obtained from the screens of NMRI and BALB/c mice shown in Table 1. We determined the colocalization of integrations in end-stage tumors with enhancers using complete (n = 6,117) and reduced (n = 2,127) integration datasets. The analyses were performed using BEDTools [53]. As control size-matched random datasets were used in iterative simulations (n = 1,000) for each analysis shown in Figure 5. In brief, the ChIP-seq datasets were annotated according to UCSC TSS annotations, excluding unmappable sequence. The median peak length and sequence content (the size in bp) of each dataset within the searched genome was then used for picking random intervals without replacement from NCBIM37/mm9. We defined direct overlaps based on extension of ChIP-seq peaks by 1,250 bp. In each case intersection was performed using 1 bp integration coordinates. The determination of empirical *p*-values based on random sampling was performed as previously described [101]. The reduced SL3-3 integration dataset was assembled by clustering integrations in bins of 2,500 bp across the genome. Clusters larger than this (containing more than two integration positions) were excluded. The position of integrations for clusters that contained more than one integration site was defined as the cluster midpoint. Out of the 2,127 coordinates in the reduced integration dataset 1,981 (93%) represented individual integration sites.

### Sequence alignment, visualization and expression analysis

FASTQ files were processed including adapter clipping and quality trimming, and aligned to the NCBI37/mm9 mouse assembly with Bowtie/TopHat [102]. The visualizations in Figures 2 and 4, as well as Additional file 1: Figures S2-S3 are based on forward read alignments for which the sequence coverage of each sample was computed strand-specifically with BEDTools [53] and scaled using Cufflinks library metrics [47]. The images were generated using the UCSC Genome Browser and post-processed using standard vector imaging tools. The remapping of *Celf2* in Figure 4E was performed using *Celf2* (chr2:6,453,742-7,029,527) and the SL3-3 proviral sequence (built using [GenBank:X00862, GenBank:AF169256]) inserted at the predetermined integration site (Additional file 2) including also the TSD. The Cufflinks transcript expression values for the loci shown in Figure 2, as well as (Additional file 1: Figures S2-S3) are available in Additional file 4.

### DNA analysis, RT-PCR, qPCR, RACE and cloning

DNA and RNA were copurified with TRIzol (Invitrogen) followed by DNA back-extraction and clean-up using QIAamp spin columns (Qiagen). To confirm integrations (Figure 1E) 40 ng of genomic DNA was subjected to 40 cycles of PCR (DreamTaq, Thermo Scientific) using three or more primer pairs designed to yield products of size differences discernible by gel electrophoresis (Additional file 1: Figure S9). In case a distinct band pattern did not appear select products were Sanger sequenced (described below). For RT-PCR and the qPCR-results shown in Figure 3 primer sequences from OriGene were used with exceptions. 500 ng of RNA was used for cDNA synthesis with the qScript cDNA SuperMix (Quanta Biosciences). PCR-reactions (DreamTaq, Thermo Scientific) were performed in a 2720 Thermo Cycler (Applied Biosystems) using standard cycling conditions, i.e. annealing at T_m_ -5°C. For qPCR measurements a Stratagene Mx3000P cycler was used (Applied Biosciences). Samples were measured in duplicates using PerfeCta SYBR Green FastMix (Quanta Biosciences) in 10 μL reactions, and beta-actin as reference. As controls, tumors were used without known integrations at the loci shown in Figure 2 and included for tumors not subjected to RNA-seq a minimum of three samples from the same mouse strain (BALB/c or NMRI) and tissue (thymus or spleen). Above-threshold values were defined as Ct values greater than 30 cycles of amplification. RACE was carried out using the SMARTer RACE cDNA amplification Kit (Clontech) in nested reactions. cDNA synthesis for 5′-RACE included random priming, and was performed according to manufacturer’s guidelines. For Sanger sequencing PCR products were cloned in the pCR4-TOPO vector using the TOPO TA Cloning Kit for Sequencing (Invitrogen). The primers used in this study are shown in Additional file 5.

## Abbreviations

MLV: Murine leukemia virus; IM: Insertional mutagenesis; LTR: Long terminal repeat; CIS: Common integration site; RNA-seq: RNA sequencing; ChIP-seq: Chromatin Immunoprecipitation with sequencing; EPU: Enhancer and promoter unit; TSS: Transcriptional start site; RTCGD: Retrovirus and Transposon tagged Cancer Gene Database; qPCR: Quantitative real-time PCR; RACE: Rapid amplification of cDNA ends; FPKM: Fragments per kilobase of transcript per million mapped reads; TSD: Target site duplication.

## Competing interests

The authors declare that they have no competing interest.

## Authors’ contribution

MS designed the deep-sequencing experiments, analyzed data and drafted the manuscript. MW carried out the splinkerette-based-PCR determination of MLV integrations. IRR and MS performed qPCR and RACE experiments. FSP conceived the study and participated in its design and direction. All authors read and consented to the contents of the manuscript.

## Supplementary Material

Additional file 1: Figure S1 Paired-end RNA-seq signatures expose retroviral integration sites. **Figure S2.** The *Tmem30b/Prkch* locus is deregulated by a bidirectional-type activation mechanism. **Figure S3.** Integration in *Wwox* induces overexpression of distal *Maf* and activation of unannotated transcription outside *Wwox*. **Figure S4.** ChIP-seq datasets from ENCODE. **Figure S5.** Integrations in end-stage tumors form clusters at immediate, intermediate and distal positions from TSSs. **Figure S6.** Chromosomal distributions of promoter-distal integrations in the complete and reduced integration datasets. **Figure S7.** Distribution of colocalizing integrations with respect to H3K4Me1 and H3K27Ac ChIP-seq peaks from spleen and thymus. **Figure S8.** Content of SL3-3 LTR-like sequence in the mouse genome assembly (NCBIM37/mm9). **Figure S9.** PCR confirmation of integrations identified in RNA sequencing. Click here for file

Additional file 2 **Integrations identified using RNA-seq.** In columns 2–3 the integrations for each tumor are numbered according to Figures 1C to E. The positions and orientations (sense, S and antisense, AS) of the integrations with respect to the reference genome, and the presence (+) or absence (−) of chimeric fusions are shown in columns 4–8. In cases the chimeric fusion point was not covered in sequencing, the integration positions correspond to the 5′-end of the murine read mates. In columns 9–12 the distance of integrations to the nearest RefSeq annotation are shown as well as the positions of proviruses (exon, intron, or outside). Columns 13–15 show if the genes have been tagged (+) or not (−) in the RTCGD or in screens of BALB/c and NMRI mice (described in the main text). The distance of the RNA-seq integrations to the nearest integrations from these screens is indicated in column 16. The four tumors (324 through 410) had also been analyzed in the NMRI mouse screen. Seven out of 13 integrations identified in the latter screen were detected with deep sequencing (Additional files 2 and 3, compared). The analyses were performed on different tumor sections. Click here for file

Additional file 3 **Integrations identified in NMRI mice using splinkerette-based PCR.** The dataset contains integrations from several cohorts of NMRI mice infected with SL3-3 including a cohort of 20 mice from which the four mouse tumors (324 through 410) subjected to RNA-seq originate (described in Methods). The integrations are sorted in order of decreasing number of tags at a locus. The first column shows tumors used in the present study for RNA-seq and/or qPCR and RACE. The layout column shows the orientations of integrations with respect to the genes: e.g. at chr5:108,167,153 the provirus is located 6,660 bp downstream from *Evi5* in the opposite (antisense) orientation relative to this gene. Click here for file

Additional file 4 **RNA-seq transcript expression values.** This table lists Cufflinks fragments per kilobase of transcript per million mapped reads (FPKM) [47] values using the UCSC mouse transcriptome prediction track for the deregulated loci described in the manuscript. The lower (conf_lo) and upper (conf_hi) bounds of the 95% confidence interval of transcript abundances is also indicated. The complete RNA-seq data is available online. Click here for file

Additional file 5 **Primer sequences.** For the DNA analyses the mouse primer sequences are available upon request. Click here for file
