# Supplementary material for: Novel principles of gamma-retroviral insertional transcription activation in murine leukemia virus-induced end-stage tumors
Source: Retrovirology. 2014 May 19;11:36. doi: 10.1186/1742-4690-11-36 (PMC4098794; doi:10.1186/1742-4690-11-36)
Supplement: Additional file 1: Figure S1 — Paired-end RNA-seq signatures expose retroviral integration sites. Figure S2. The Tmem30b/Prkch locus is deregulated by a bidirectional-type activation mechanism. Figure S3. Integration in Wwox induces overexpression of distal Maf and activation of unannotated transcription outside Wwox. Figure S4. ChIP-seq datasets from ENCODE. Figure S5. Integrations in end-stage tumors form clusters at immediate, intermediate and distal positions from TSSs. Figure S6. Chromosomal distributions of promoter-distal integrations in the complete and reduced integration datasets. Figure S7. Distribution of colocalizing integrations with respect to H3K4Me1 and H3K27Ac ChIP-seq peaks from spleen and thymus. Figure S8. Content of SL3-3 LTR-like sequence in the mouse genome assembly (NCBIM37/mm9). Figure S9. PCR confirmation of integrations identified in RNA sequencing. [file 1742-4690-11-36-S1.pptx]

## Slide 1
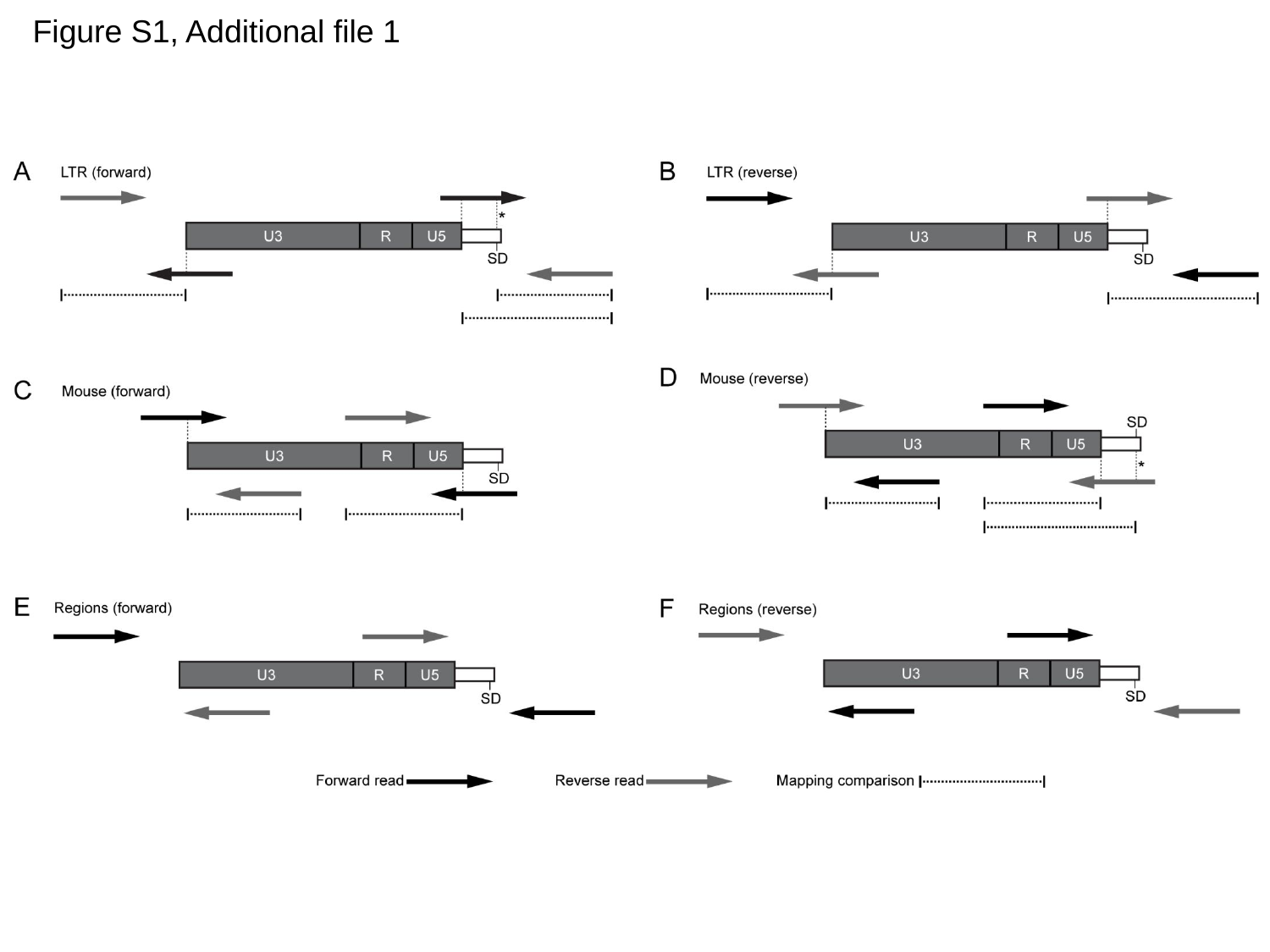

Figure S1, Additional file 1

## Slide 2
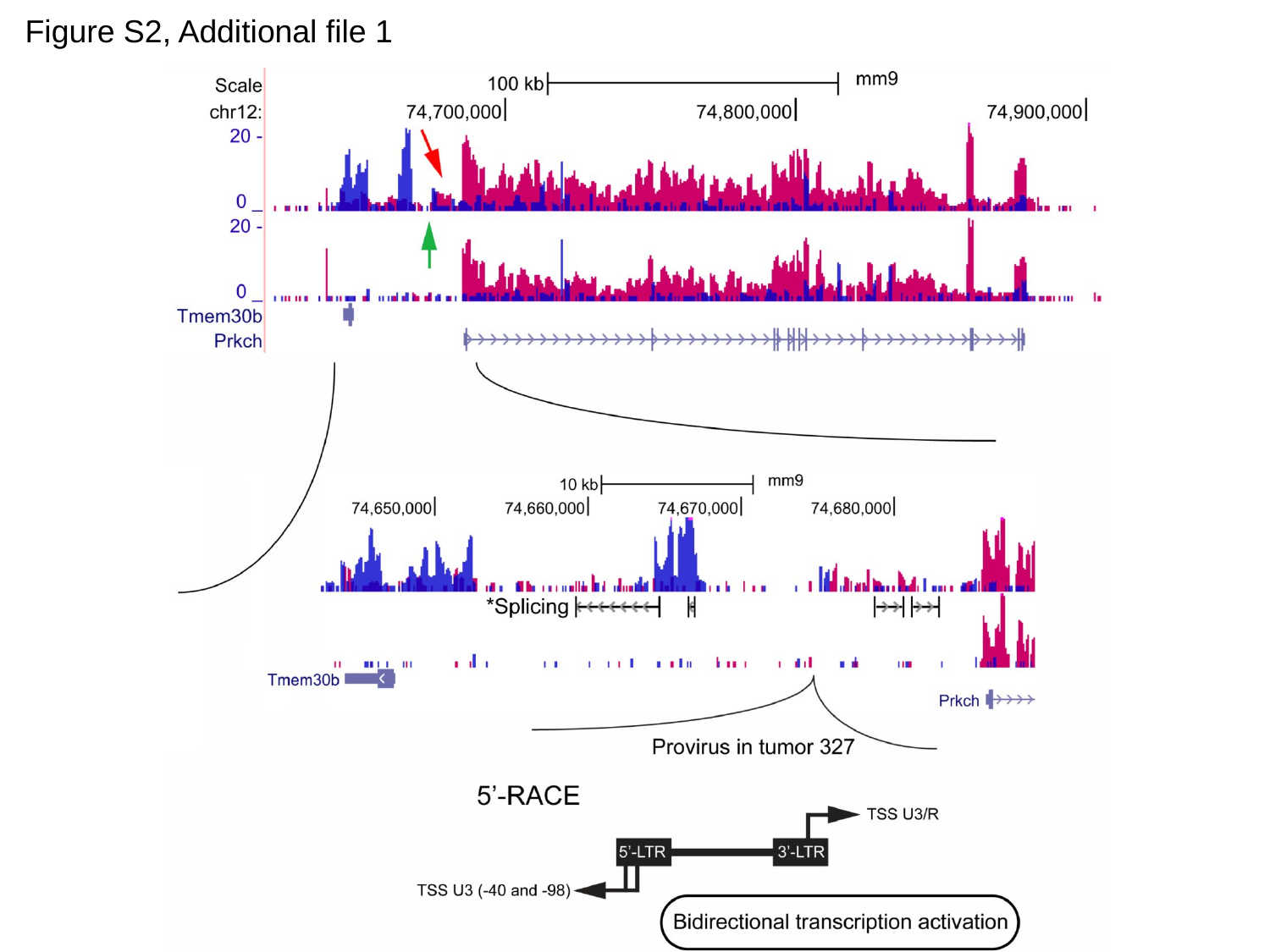

Figure S2, Additional file 1

## Slide 3
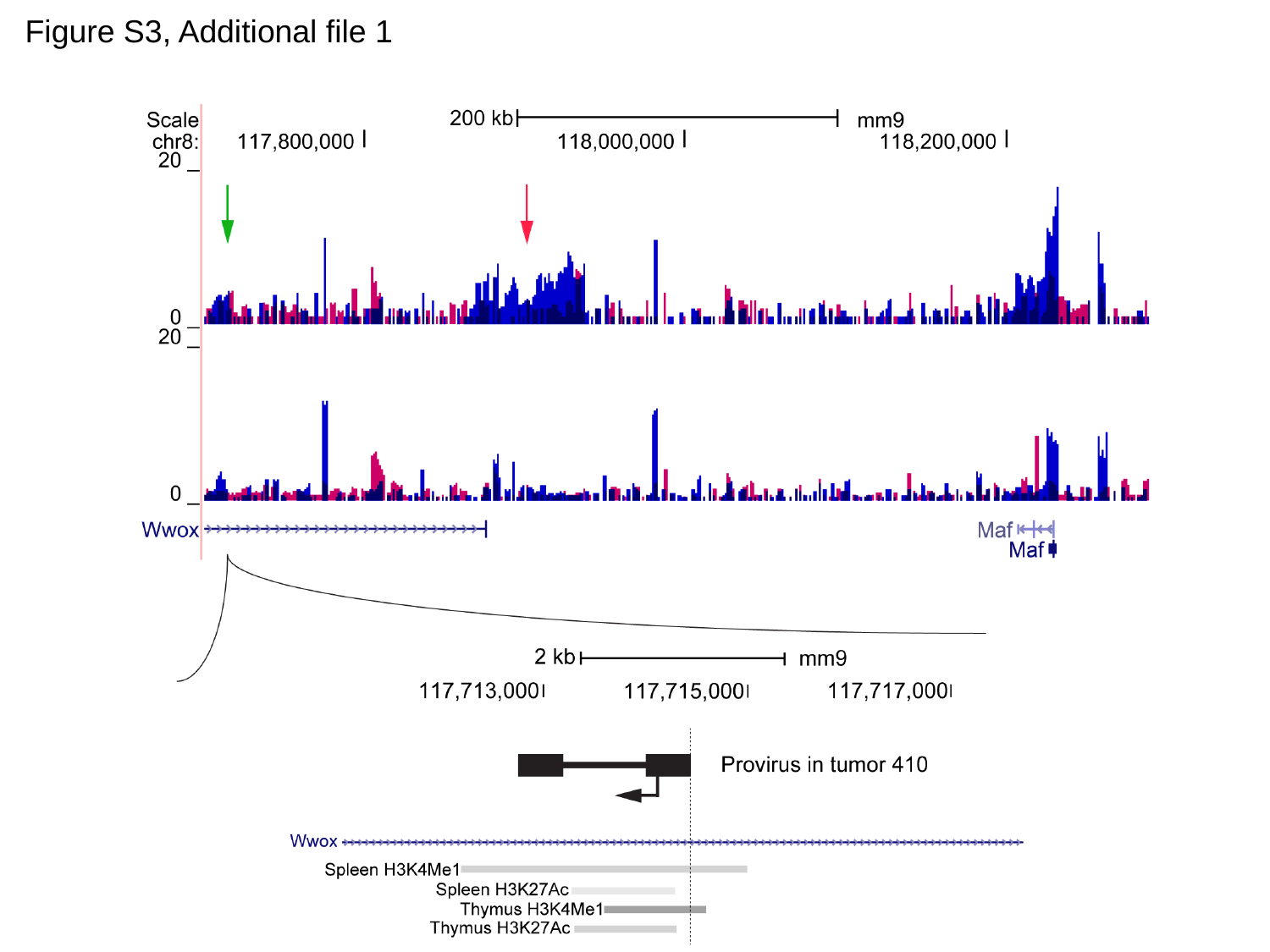

Figure S3, Additional file 1

## Slide 4
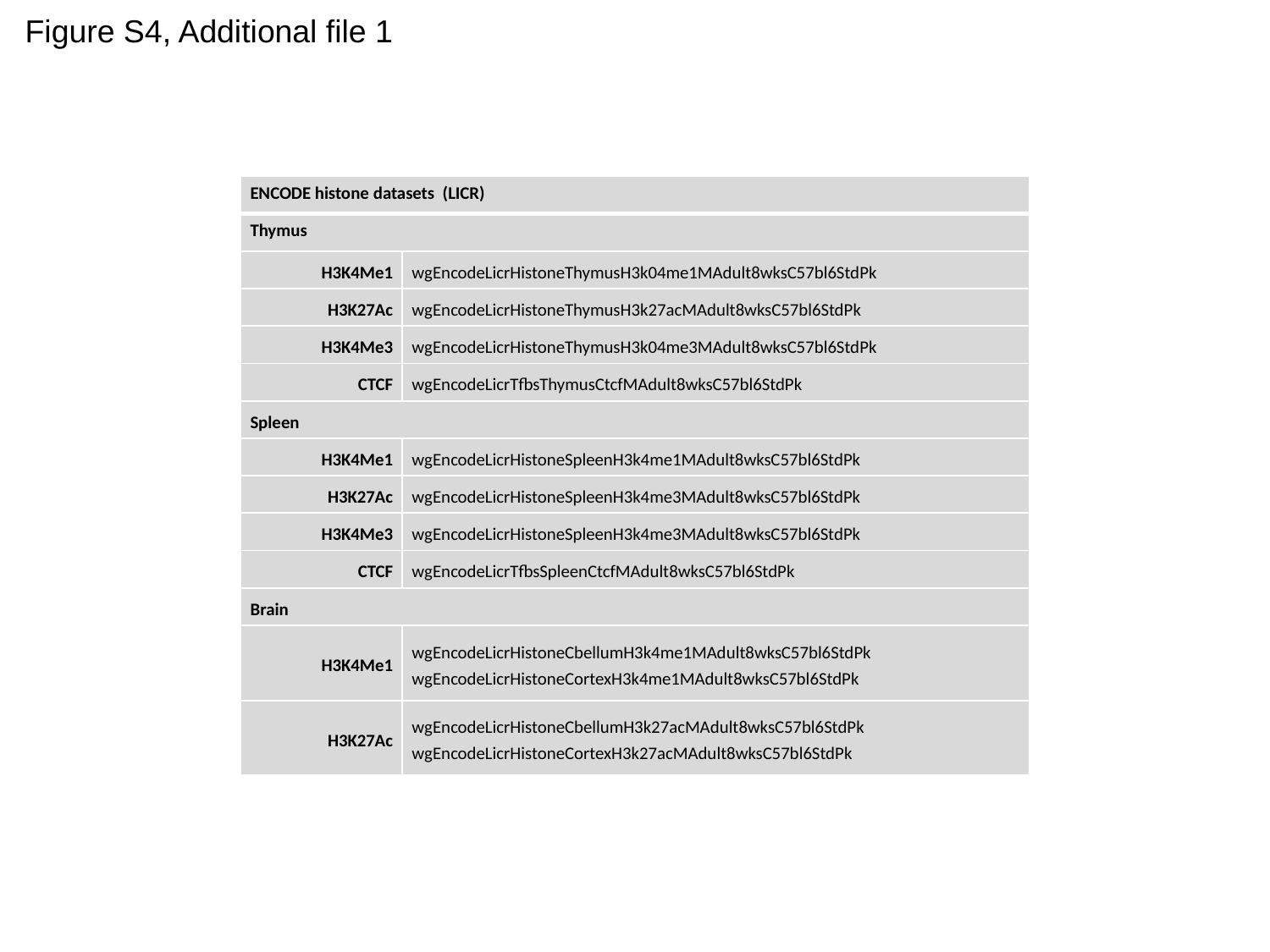

Figure S4, Additional file 1
| ENCODE histone datasets (LICR) | |
| --- | --- |
| Thymus | |
| H3K4Me1 | wgEncodeLicrHistoneThymusH3k04me1MAdult8wksC57bl6StdPk |
| H3K27Ac | wgEncodeLicrHistoneThymusH3k27acMAdult8wksC57bl6StdPk |
| H3K4Me3 | wgEncodeLicrHistoneThymusH3k04me3MAdult8wksC57bl6StdPk |
| CTCF | wgEncodeLicrTfbsThymusCtcfMAdult8wksC57bl6StdPk |
| Spleen | |
| H3K4Me1 | wgEncodeLicrHistoneSpleenH3k4me1MAdult8wksC57bl6StdPk |
| H3K27Ac | wgEncodeLicrHistoneSpleenH3k4me3MAdult8wksC57bl6StdPk |
| H3K4Me3 | wgEncodeLicrHistoneSpleenH3k4me3MAdult8wksC57bl6StdPk |
| CTCF | wgEncodeLicrTfbsSpleenCtcfMAdult8wksC57bl6StdPk |
| Brain | |
| H3K4Me1 | wgEncodeLicrHistoneCbellumH3k4me1MAdult8wksC57bl6StdPk wgEncodeLicrHistoneCortexH3k4me1MAdult8wksC57bl6StdPk |
| H3K27Ac | wgEncodeLicrHistoneCbellumH3k27acMAdult8wksC57bl6StdPk wgEncodeLicrHistoneCortexH3k27acMAdult8wksC57bl6StdPk |

## Slide 5
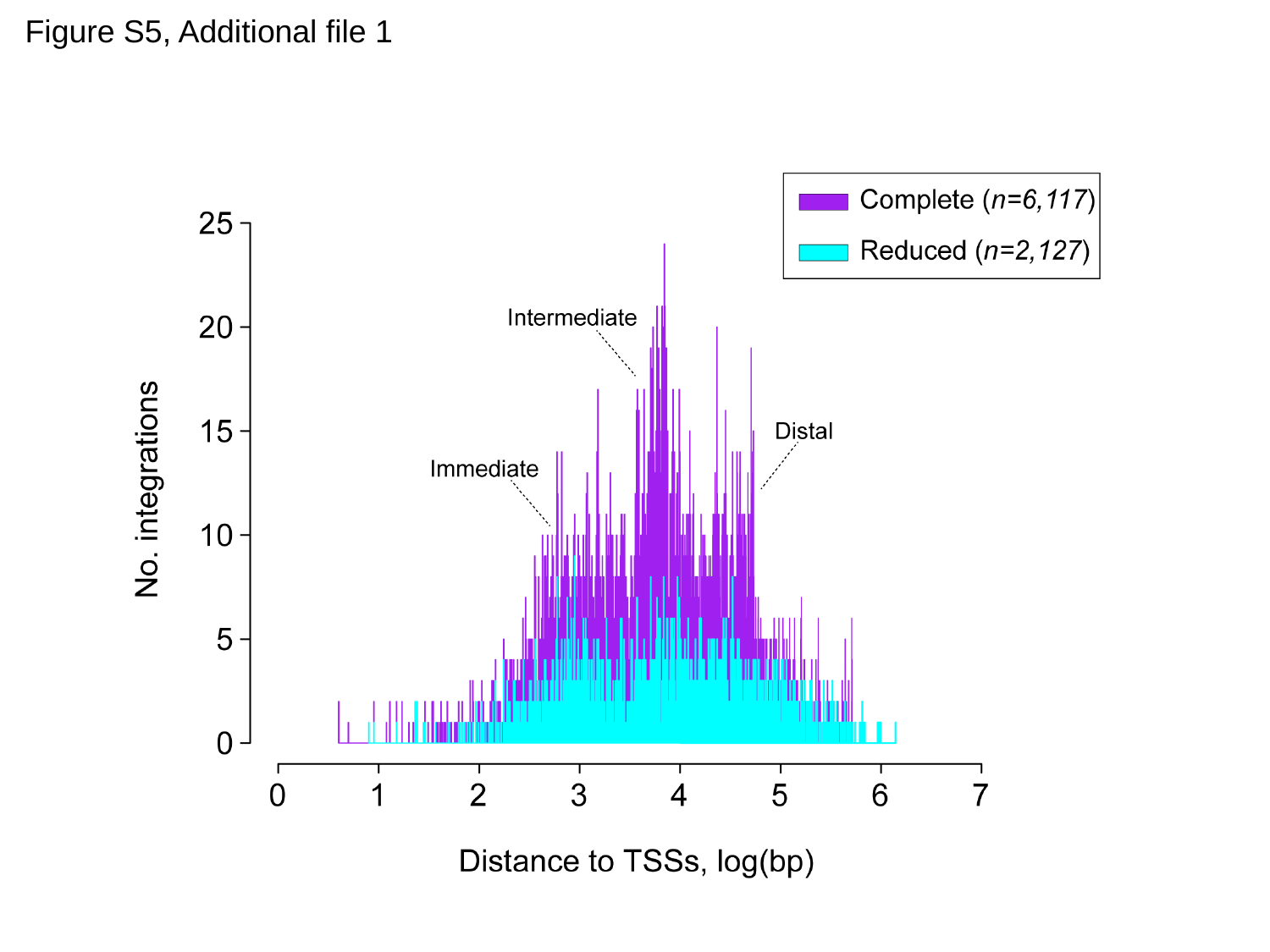

Figure S5, Additional file 1

## Slide 6
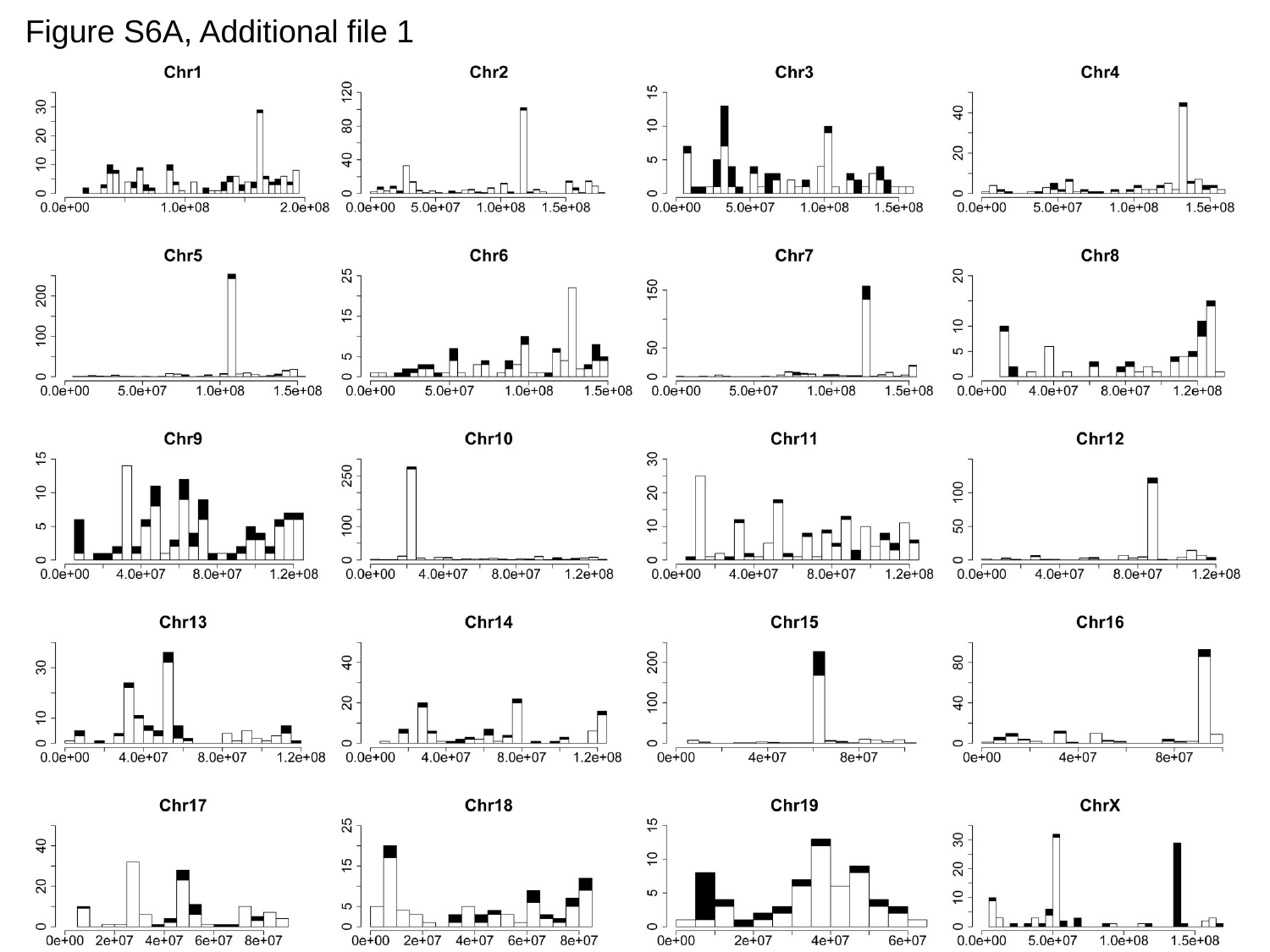

Figure S6A, Additional file 1

## Slide 7
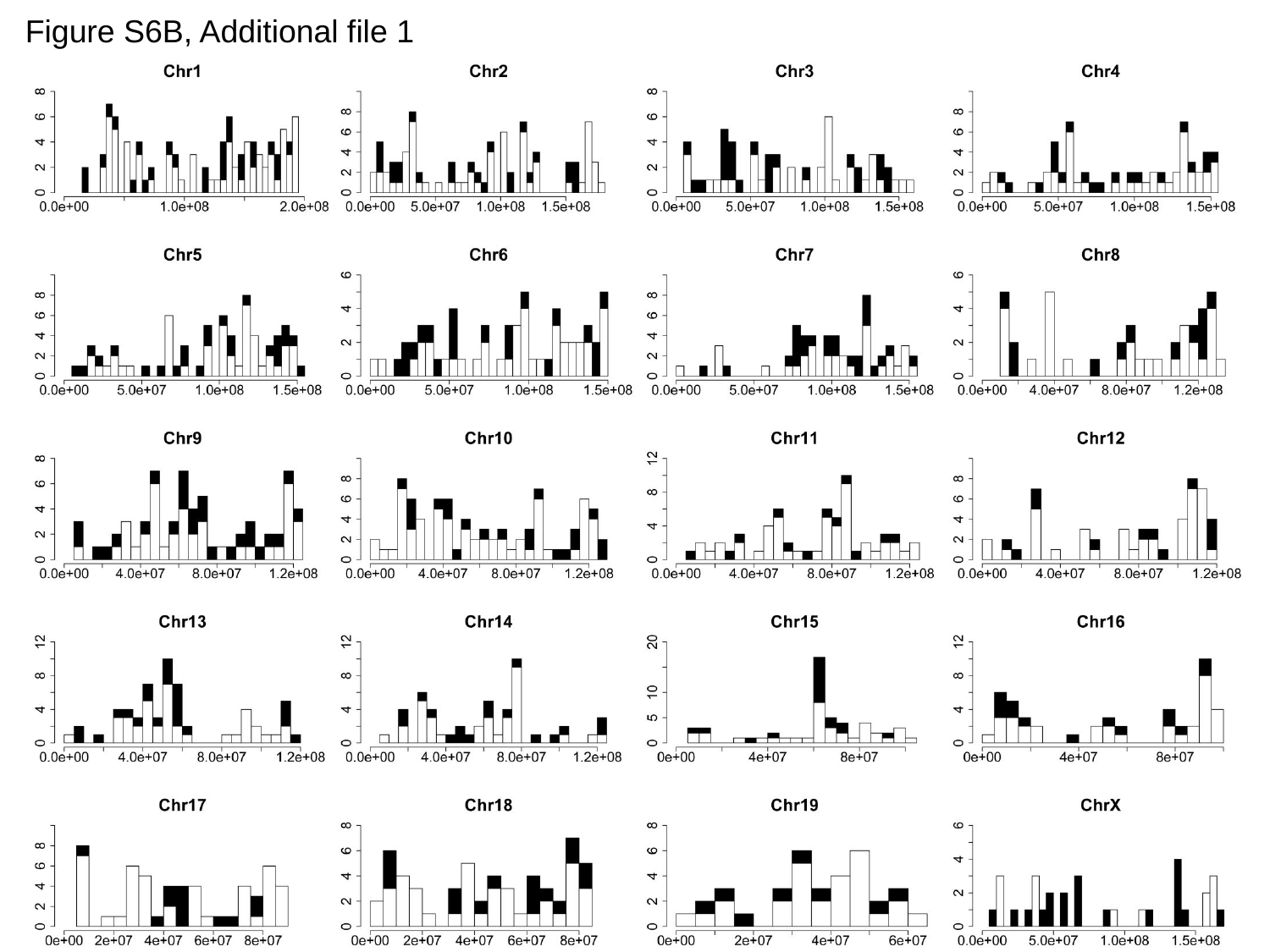

Figure S6B, Additional file 1

## Slide 8
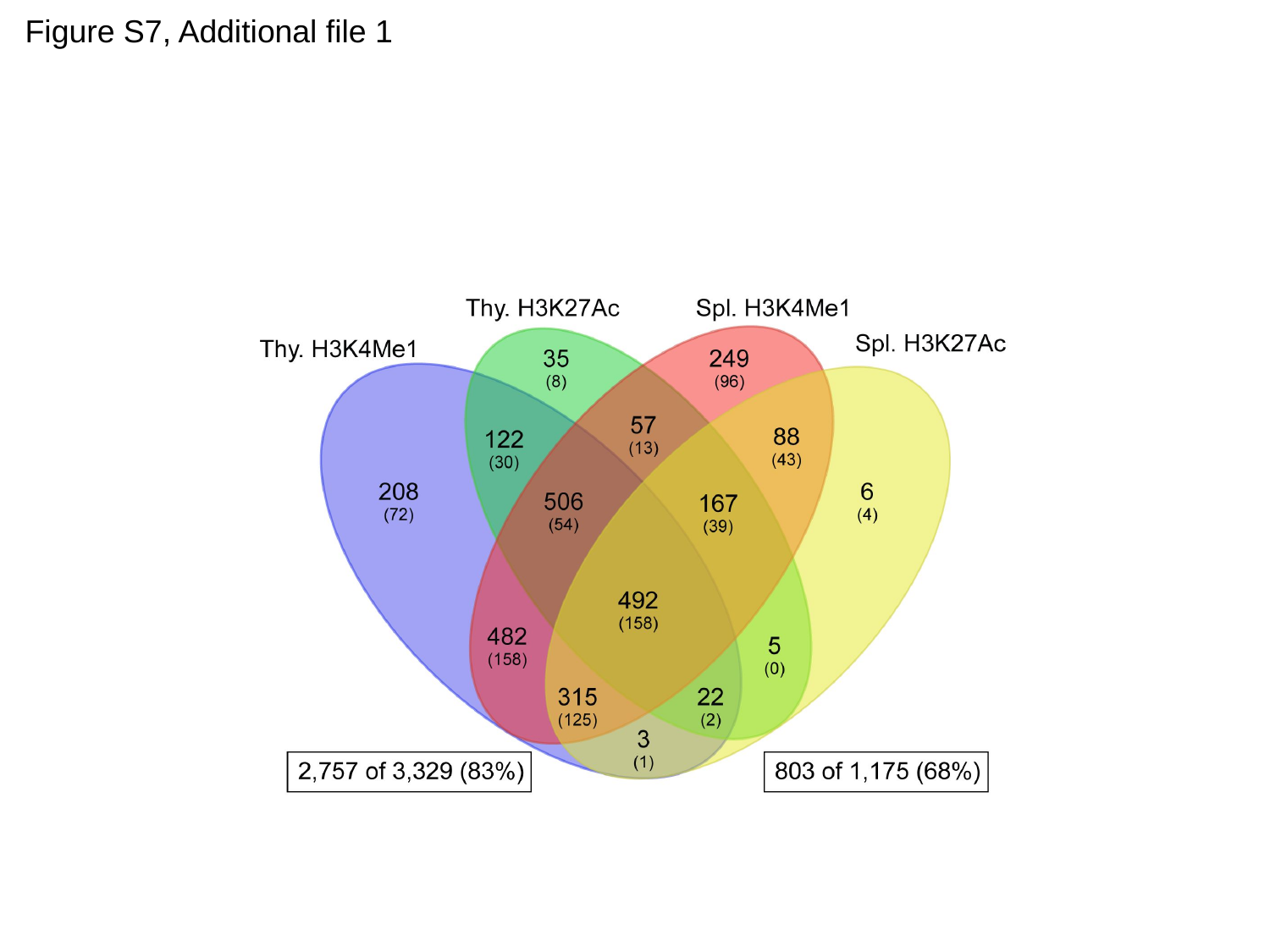

Figure S7, Additional file 1

## Slide 9
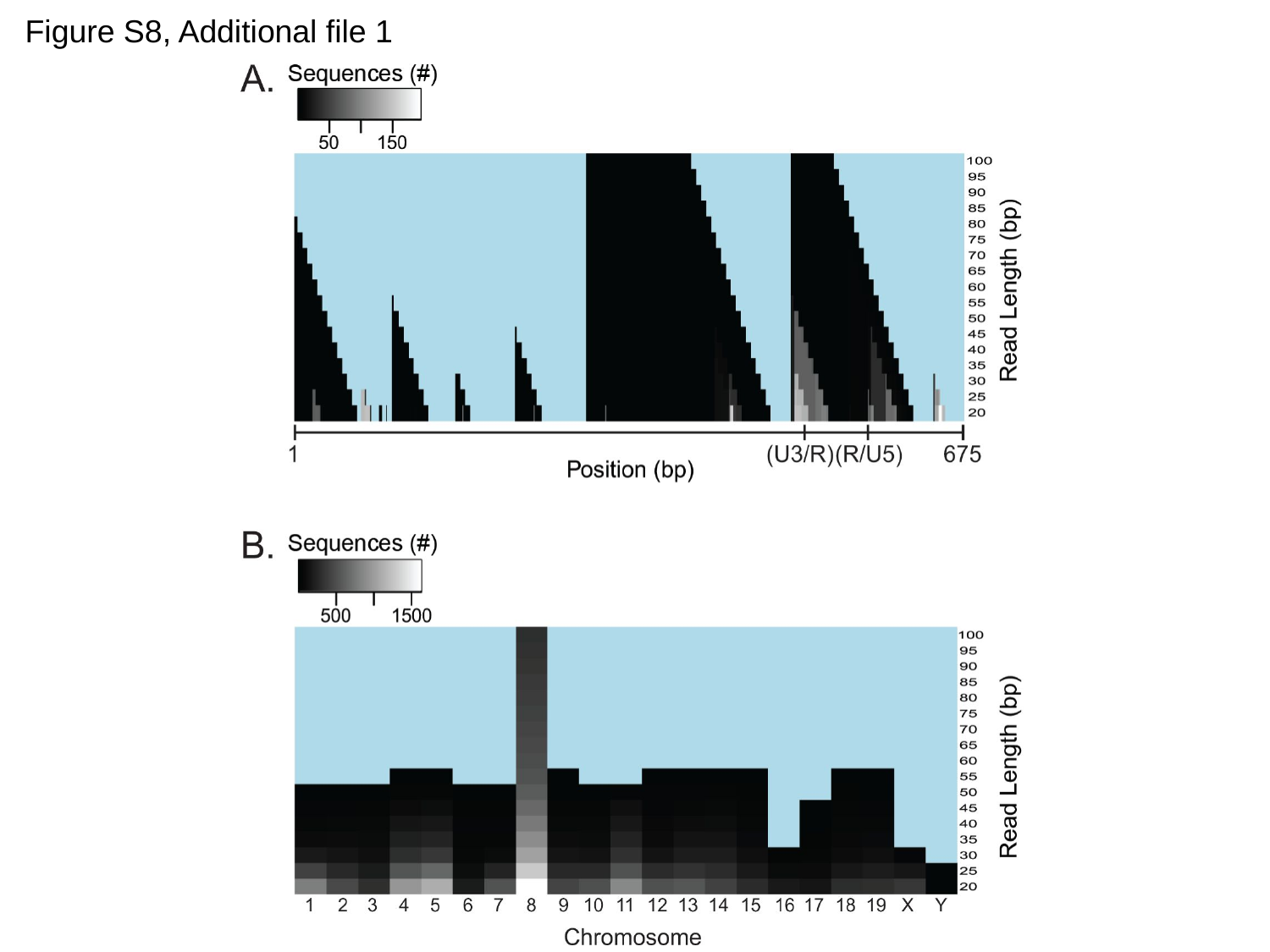

Figure S8, Additional file 1

## Slide 10
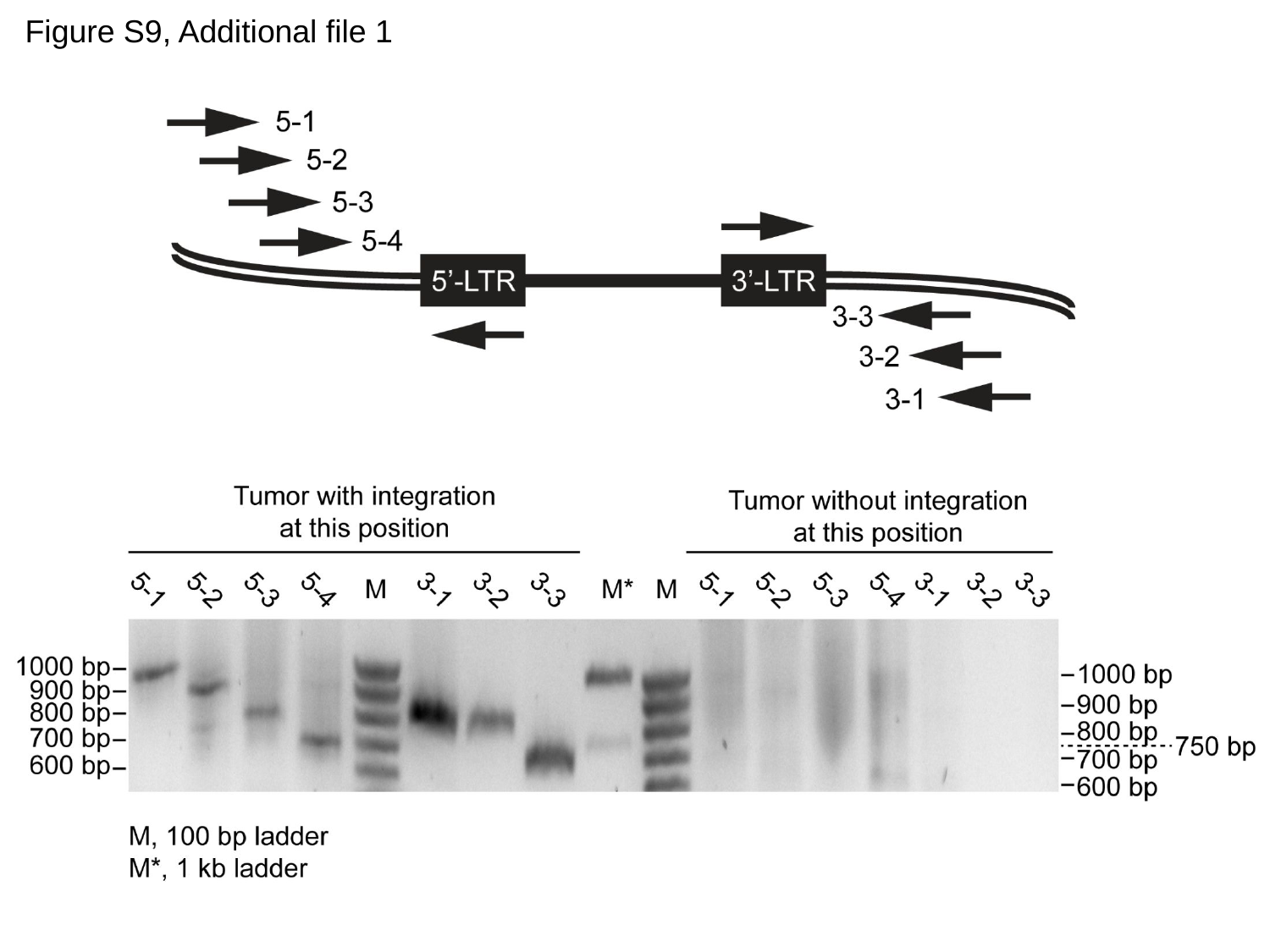

Figure S9, Additional file 1
