# Supplementary material for: Novel principles of gamma-retroviral insertional transcription activation in murine leukemia virus-induced end-stage tumors
Source: Retrovirology. 2014 May 19;11:36. doi: 10.1186/1742-4690-11-36 (PMC4098794; doi:10.1186/1742-4690-11-36)
Supplement: Additional file 5 — Primer sequences. For the DNA analyses the mouse primer sequences are available upon request. [file 1742-4690-11-36-S5.docx]

**Additional file 5**

| ***qPCR and RT-PCR*** | **Primers (forward/reverse)** |
| --- | --- |
| *Klf7* downstream region | 5TCTGCAGAAAGGAAGGCTGACA3/5ACACCTTCCCAACCGACGTG3 |
| *Klf7* | 5GGAAGGATGCGAGTGGCGTTTT3/5CGCAAGATGGTCAGACCTGGAG3 |
| *Syn2* | 5CCTGCTCTGAAATGTTTGGTGGC3/5TCTGTCCTCCACTTGGTGTTCC3 |
| Ak038749 | 5ACCTTCGTGGTTTCAAGAGGAGA3/5TTGGGATTCTGGTGTCGTGCACC3 |
| *Timp4* (1) | 5CCCTCTGTGGTGTGAAGCTAGAAACC3/5CATAGCAAGTGGTGATTTGGCAGC3 |
| *Timp4* (2) | 5CCACTCGGCTCTAGTGATACGGG3/5GGTTTCTAGCTTCACACCACAGAGGG3 |
| *Timp4* (3) | 5AGCAAAGACCCTGCTGACACTC3/5ACAGAGGGAAGAGTCAAATGGCG3 |
| *Col4a5* | 5GGCATACAAGGTGTGGCAGGAA3/5ACCATCTCTGCCTGGACTACCT3 |
| *Irs4* | 5CGTATGTGCCAATGAGACCAGG3/5GTGGCGTTTTGTTGAAGCAGAGG3 |
| *Celf2* (3’-end) | 5CCTCTGTCTAGCACAAGCAGTG3/5CAGCCAATCCTTGTAGAGTCCC3 |
| *Celf2* (upstream intron) | 5TCAGGTGAATCAGACTCCTAATTTC3/5GTGAAGCAGGAATTATGAAGGACTA3 |
| *Ccr9* | 5GCCATGTTCATCTCCAACTGCAC3/5CCTTCGGAATCTCTCGCCAACA3 |
| *Slc6a20a* | 5TGCCAGCATTGTCACCTTCTCC3/5GGTTGCTGACTGTCAGAAAGCC3 |
| *Slc6a20b* | 5GCTAACACTGGGTATGGGAAGC3/5TGCCAATGGCACAGTTGAGGAG3 |
| *Lztfl1* | 5AACGGTAGATTCCTGCTTTCAGG3/5TCGGACTCCACCTCACTGTGTA3 |
| **RACE** | **Primers (primary/secondary)** |
| Ak038749 | 5GGCTGAAGGGGATCTGGGCAAAGAG3/5GGACCCAGAGCAGCAATGGCAAGAA3 |
| *Klf7* downstream region | 5TTTGCATCTGCCGCACAGGGATTAG3/5CCAAGTAGGTGCTGTCCCCGTGAGA3 |
| *Celf2* (promoter insertion) | 5CTCAGCCCATCAGGTCCCCGGAG3/5CTCTTCTGGGCATATTGAAAGACCC3 |
| *Celf2* (upstream transcript.) | 5CTCAGCCCATCAGGTCCCCGGAG3/5GCAGTGCATTCTGGGCCTCAAGTGC3 |
| *Celf2* (3-’end) | 5CTCCGGGGACCTGATGGGCTGAG3 |
| *Prkch/Tmem30b* (5-LTR) | 5CGCTGGAGCCGTATCACTGCCTGCC3/5CACTGCCTGCCACCCGTTATC3 |
| *Prkch/Tmem30b* (3-LTR) | 5GACGACACGGCATGGGAATACAGAA3 |
| **DNA analysis (host primer sequences are not shown)** | |
| LTR sense | 5TGAAAGACCCCTTCATAAGGCTTAG3 |
| LTR antisense | 5TGAAAGACCCCCAGGCTGGGCAG3 |
